# Q-Learning-Driven Butterfly Optimization Algorithm for Green Vehicle Routing Problem Considering Customer Preference

**DOI:** 10.3390/biomimetics10010057

**Published:** 2025-01-15

**Authors:** Weiping Meng, Yang He, Yongquan Zhou

**Affiliations:** 1 College of Artificial Intelligence, Guangxi Minzu University, Nanning 530006, China; 2 Guangxi Key Laboratories of Hybrid Computation and IC Design Analysis, Nanning 530006, China

**Keywords:** butterfly optimization algorithm (BOA), Q-learning, benchmark functions, global optimization, green vehicle routing problem with time windows (GVRPTW), metaheuristic

## Abstract

This paper proposes a Q-learning-driven butterfly optimization algorithm (QLBOA) by integrating the Q-learning mechanism of reinforcement learning into the butterfly optimization algorithm (BOA). In order to improve the overall optimization ability of the algorithm, enhance the optimization accuracy, and prevent the algorithm from falling into a local optimum, the Gaussian mutation mechanism with dynamic variance was introduced, and the migration mutation mechanism was also used to enhance the population diversity of the algorithm. Eighteen benchmark functions were used to compare the proposed method with five classical metaheuristic algorithms and three BOA variable optimization methods. The QLBOA was used to solve the green vehicle routing problem with time windows considering customer preferences. The influence of decision makers’ subjective preferences and weight factors on fuel consumption, carbon emissions, penalty cost, and total cost are analyzed. Compared with three classical optimization algorithms, the experimental results show that the proposed QLBOA has a generally superior performance.

## 1. Introduction

With the ongoing increase in market size, the logistics and transportation business’s position in social production activities has become more prominent. With the spread of the notion of green environmental protection, public awareness of greenhouse gas emissions in transportation has grown. However, in pursuing cost-cutting and efficiency improvements, logistics companies frequently overlook the possible environmental impact of their operations. Carbon and nitrogen oxide emissions, in particular, contribute to global climate change while also endangering human health and ecosystems. Thus, encouraging fuel-efficient vehicles and other emission-reducing technologies is essential to achieving the country’s low-carbon objective. In the area of urban distribution, daily traffic congestion in urban areas has a direct impact on the fuel consumption and carbon emissions of vehicles; in turn, this congestion state will raise vehicle fuel consumption and emissions, which will have an indirect impact on the financial and environmental costs associated with transportation [1].

The vehicle routing problem (VRP) refers to a distribution center providing services to customers with goods in demand and meeting their needs according to established routes within a specific range in order to achieve the goals of minimum cost, shortest distance, and minimum time consumption. The VRP seeks to minimize the total cost and trip distance. The green vehicle routing problem (GVRP) is a developing study area. It is defined by the optimization aims of lowering operating costs, reducing energy consumption and carbon emissions, and ensuring customer happiness while adhering to customer service needs and vehicle capacity limits. Economic and environmental benefits can be optimized by strategically planning vehicle departures, times, and routes [2]. The GVRP is a variation of the classic vehicle routing problem (VRP) that tries to lessen the environmental impact, such as fuel usage and carbon emissions generated by the allocation process. The VRP problem with time windows (VRPTW) considers customer demand and demands customer service be completed within a given period. Researchers have classified the GVRP as an NP-hard issue. Several academics have looked into the use of the GVRP to partially reduce fuel usage and carbon emissions. Metaheuristics were applied to overcome the fuel consumption problem. Internationally, research on the GVRP has yielded outstanding results. For instance, the pollution-routing problem (PRP), which considers the journey distance, greenhouse gas emissions, and travel time expenses, was first presented by Bektas et al. with the primary goal of lowering fuel consumption when a vehicle is operating. Furthermore, PRP mathematical models with and without time windows in mixed-integer form were established [3]. A thorough emission model was presented by Barth et al. to determine vehicle fuel usage [4]. Demir and Bektas, drawing from the work of Bektas et al., examined and contrasted a number of widely used emission models pertaining to fuel consumption and greenhouse gas emissions associated with road freight transportation, and then contrasted the model output with data from real-world road usage [5]. Laporte proposed an adaptive large neighborhood search algorithm to solve the PRP. It employed both novel and pre-existing deletion and insertion strategies to optimize the algorithm and enhance the quality of the solution, and a large number of experiments were conducted to confirm its efficacy [6]. To solve the multi-depot GVRP problem, Mehlawat et al. suggested a hybrid genetic algorithm and conducted experiments to confirm the technique’s robustness [7]. Previous studies have shown that carbon dioxide (CO_2_) emissions are directly proportional to fuel consumption, and this relationship is significantly affected by the speed of vehicles. Franceschetti et al. first proposed the time-dependent pollution-routing problem, which extended the PRP problem by explicitly considering traffic congestion scenarios. The integer linear programming formulation of the TDPRP has also been described in detail [8]. Cimen et al. proposed a heuristic algorithm based on approximate dynamic programming to solve the time-dependent green vehicle routing problem (TDGVRP) considering the time dependence and randomness of vehicle speeds in time-varying road networks [9]. Kazemian et al. took greenhouse gas emissions and fuel consumption as the optimization objectives of the TDGVRP and reduced the complexity of the solution by transforming the problem with time windows into a problem without time windows so as to reduce carbon emissions and fuel consumption while controlling the total cost [10]. Rui Qi employed the QMOEA algorithm, which is based on Q-learning, to address the time-dependent green vehicle routing problem with time windows (TDGVRPTW) [11]. With carbon emissions as the minimum objective, Prakash et al. established a GVRP model with time window constraints [12]. Wang et al. proposed a bi-objective model to minimize total carbon emissions and operating costs and simultaneously implemented piecewise penalty costs for early and late arrivals to reduce waiting times and improve customer satisfaction, which was solved by the multi-objective Particle Swarm Optimization (MOPSO) algorithm [13]. Wu et al. set the sum of the driving cost, fuel consumption cost, time window penalty cost, and fixed vehicle usage cost as the optimization objective and established a time-varying GVRP model with vehicle capacity and time window constraints [14]. Zhang et al. established a mixed-integer linear programming model considering time-varying traffic conditions, customer time windows, and vehicle energy consumption functions and solved it using several different metaheuristic algorithms, which is convenient for practical application [15].

Based on a complete understanding of the environmental and logistics system properties of urban areas, mathematical models are developed with the objective of minimizing the cost; the total cost includes the fixed cost of vehicles, fuel consumption cost, and carbon emission cost, as well as the penalty cost. This section explores the environmental challenges faced by road freight transport operators and proposes a systematic optimization approach. Without loss of generality, the improved butterfly optimization algorithm (BOA) is combined with Q-learning. The effectiveness of the proposed method is verified by several computational experiments. The results show that the proposed method effectively reduces the total allocation cost, reduces carbon emissions and fuel consumption, and avoids daily traffic congestion.

The butterfly optimization algorithm (BOA) is a novel metaheuristic optimization algorithm first proposed by Arora and Singh [16]. Smell is the sense that butterflies value most. The BOA offers the advantages of effectiveness and simplicity. The two stages of butterfly actions are separated by simple updating algorithms for both local search and global movement [17]. Moreover, the BOA can address issues instantly and requires fewer control parameters [18]. The algorithm’s advantages have spurred other academics to develop it. In order to address the problem of premature convergence, Elhoseny et al. used a COVID-19 dataset to assess the suggested approach after applying the BOA to a hybrid feature selection model [19]. The Flower Pollination Algorithm (FPA) and BOA were merged by Zhou et al. [20]. The multi-swarm binary BOA (MBBOA) was proposed by Nader et al. for the 0-1 MKP issue [21]. Mazaheri H. et al. introduced the concept of an intelligent throwing agent and proposed an efficient routing algorithm based on the BOA to calculate the shortest path and minimize the power of obstacle avoidance UAVs [22]. Chatterjee S. et al. proposed a population-based metaheuristic BOA to predict the secondary structure of ribonucleic acid (RNA) [23]. Bhanja S et al. proposed a fuzzy time-series optimization based on the BOA (FTSBO) algorithm to optimize all hyperparameters of the Type-2 fuzzy time-series (FTS) prediction method [24]. Alhassan A. M. et al. proposed a Gaussian Kernel Chaotic Butterfly Optimization Algorithm (TCBOAGK) to realize the automatic detection of affected areas in X-ray images [25]. Manickam S. et al. used the improved BOA (IBOA) for feature optimization and realized a convolutional neural network (CNN) fused with a long short-term memory network (LSTM) for voice identification [26].

Even though numerous academics have expanded the BOA procedure, it still has certain shortcomings. The traditional BOA cannot fully balance the global exploration and local exploitation in the iterative process and has the shortcomings of easily falling into local optima and having low solution accuracy and slow convergence speed [27,28]. To more effectively maintain the exploration and exploitation properties’ coordination and increase the optimization ability to avoid slipping into a local optimum, the BOA paired with Q-learning was developed, and this combination is known as the QLBOA.

The following is a summary of the principal achievements:

(1) The BOA was fused with reinforcement learning, the reinforcement learning mechanism was introduced, and the butterfly update strategy was fused with dynamic Gaussian mutation. The variance of random state–action pairs and Gaussian random numbers were used to balance the exploration and exploitation of the algorithm.

(2) Species migration and mutation strategies were introduced to enhance the population diversity of the algorithm.

(3) Eighteen challenging benchmark functions in high dimensions and the CEC2022 test suite were chosen for the preliminary detection of several performance characteristics, including accuracy, convergence, and statistics.

(4) The green vehicle routing problem with time windows that take into account consumer needs is solved using the QLBOA. This study examines the impact of decision makers’ subjective preferences and weight variables on various optimization targets, the overall cost, and the comparison with other traditional metaheuristic algorithms.

This is how the rest of the paper is organized: A brief introduction to the BOA, Q-learning, and the adaptive Gaussian mutation mechanism is provided in Section 2. The QLBOA is thoroughly introduced in Section 3. Section 4 provides an in-depth examination of the initial simulation outcomes for reference functions. The integration of the suggested method for the green vehicle routing problem with consumer-focused time frames is shown in Section 5. Conclusions and future works are presented in Section 6.

## 2. Hybrid Mechanism Butterfly Optimization Algorithm

### 2.1. Butterfly Optimization Algorithm

Simon Arora and Singh presented the initial proposal for the BOA [16]. As one of their most vital senses, smell is thought to be able to both assist butterflies in finding food and serve as a useful means of communication between them. Every butterfly has the capacity to emit a distinct scent that is associated with its fitness rating, according to the BOA. Put differently, the butterfly’s fitness changes with movement. The butterfly uses a technique called global search to locate another butterfly if it detects a stronger scent from that butterfly. The butterfly goes into a stage known as local search, where it moves randomly in an attempt to find more scents.

The principle of butterfly aroma generation is as follows:

(1) $$f_{i}=cI^{a}$$

where $f_{i}$ defines the scent intensity function; c represents the sensory morphology coefficient; I represents the stimulus intensity, which is the function’s fitness value; and a is a random number and denotes the intensity coefficient in the [0, 1] range.

The global search phase is shown in Equation (2):

(2) $$x_{i}^{t+1}=x_{i}^{t}+\left(r^{2}\times g^{*}-x_{i}^{t}\right)\times f_{i}$$

where $x_{i}$ signifies the solution vector for the ith person in the *t*th iteration, *r* is a random number in [0, 1], $g^{*}$ represents the optimal solution among all solutions in the current iteration, and $f_{i}$ represents the amount of scent created by the *i*th individual.

When a butterfly cannot identify the scent of another butterfly inside the search region, it will act haphazardly. We refer to this stage as the local search phase. The local search phase makes use of the following mathematical model:

(3) $$x_{i}^{t+1}=x_{i}^{t}+\left(r^{2}\times x_{j}^{t}-x_{k}^{t}\right)\times f_{i}$$

where $x_{j}$ and $x_{k}$ represent the solution vectors of the jth and kth individuals, respectively. The global optimization and local search procedures are governed by a fixed switching probability, $p\in \left[0,1\right]$.

(4) $$c^{t+1}=c^{t}+\frac{0.025}{c^{t}\times \max_{\mathrm{iter}}}$$

The pseudocode for the BOA is displayed as follows Algorithm 1:
**Algorithm 1** BOAInitialize parameters and generate the initial population of N butterflies.Calculate the fitness and choose the best solution.**While** stopping criteria are not met, **do**      **For** each butterfly in the population, **do**            Generate fragrance using Equation (1).      **End for**      Calculate the fitness and choose the best individual.      **For** each butterfly in the population, **do**            Set *r* in [0, 1] randomly.            **If**
*r* < *p*, then                  Update position using Equation (2)            **Else**                  Update position using Equation (3).            **End if**      **End for****End while**Output the best solution.

### 2.2. Q-Learning

One of the best techniques for reinforcement learning is Q-learning, which Watkins [29] introduced. Reinforcement learning, sometimes referred to as evaluation learning and reinforcement learning, is a significant machine learning technique with several applications in the domains of analysis, prediction, and intelligent control robots [30]. The foundation of Q-learning is the idea of reward and punishment, whereby the environment provides the learner with an appropriate response as soon as a state shifts. The current state changes to the subsequent state upon completion of an activity. The Q-table, which is represented as Q(s, a), where *s* is the state and a is the action, indexes a Q-value as the cumulative reward using a state–activity pair. With respect to a particular state–action pair, the reward/penalty is dynamically updated in the Q-table. Temporal difference learning includes Q-learning. The algorithm simulates an episode, integrates dynamic programming and the Monte Carlo MC algorithm [31], and then estimates the value of the state prior to execution based on the value of the new state following one or more action steps.

To represent the update of a Q-table in Q-learning, we use the following formula:

(5) $$Q_{\left(t+1\right)}(S_{t},a_{t})=Q_{\left(S_{t},a_{t}\right)}+a_{t}[r_{t}+\gamma \times \max(Q_{t}(S_{t+1},a_{t+1}))-Q_{t}(S_{t},a_{t})]$$

where α is the discount factor within [0, 1], *r* is the reward/penalty, and the value is given in Equation (6). The learning rate *γ* varies within [0, 1], and the value is shown in Equation (7).

(6) $$r_{t}=\left\{\begin{array}{ll} 1 & \mathrm{reward}\\ -1 & \mathrm{punish} \end{array}\right.$$

(7) $$\alpha =1-0.9\times \frac{t}{MaxIter}$$

Exploration or exploitation is to be performed according to parameter *α*’s value. All defined states are investigated if the value is near 1, as this indicates that the most recent data were obtained with greater priority. Conversely, the present data are given more weight if the value is near 0. We put the value at 0.8 [32].

### 2.3. The Adaptive Gaussian Mutation Mechanism

**Figure 1:**
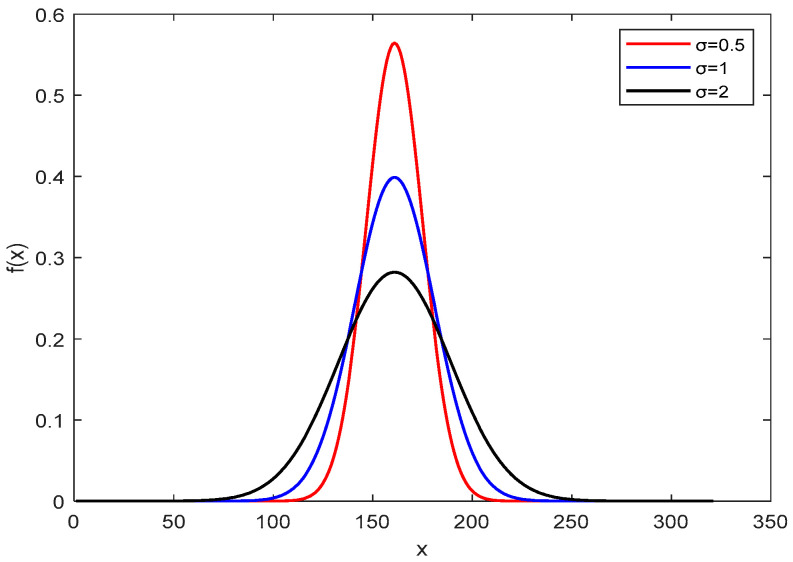
Result of the effect of standard deviation on the function.

The value of the parameter determines whether exploration or exploitation will be carried out. If the value is close to one, then the most recent data are used in the first iteration of the algorithm, which is required to explore a wide range, and in the second iteration, it is necessary to increase the optimization accuracy and avoid premature optimization. As a result, the variance of a Gaussian random number can be utilized to balance algorithm exploration with development. The figure shows that the chance of the function’s value being at the mean is inversely related to the amount of variance. In the early iteration of the algorithm, a large value is assigned according to Equation (8) so that the butterfly can explore a large range. In the later iteration, the value is adaptively reduced so that the butterfly search range is more accurate, the optimization accuracy is enhanced, and the local optimum is avoided. The effect of the standard deviation on the function is shown in Figure 1.

The Gaussian probability density function can be expressed as follows:

(8) $$f(x)=\frac{1}{\sqrt{2\pi \delta }}e^{\frac{{\left(x-\mu \right)}^{2}}{2\delta^{2}}}$$

where μ is the mean value, and δ is the standard variance.

(9) $$\delta (t)=\delta_{\max}-\frac{(\delta_{\max}-\delta_{\min})\times Iter_{current}}{Iter_{\max}}$$

where $\delta_{\max}$ is the maximum value of variance, $\delta_{\max}$ is the minimum value, ${Iter}_{current}$ is the current iteration number, and ${Iter}_{\max}$ is the maximum iteration number.

## 3. Butterfly Optimization Algorithm with Q-Learning (QLBOA)

Due to the early convergence and tendency to fall into the local optimum of the classic BOA, this section suggests an improved BOA based on reinforcement learning. The population update in the first iteration updates each individual position using the two fundamental BOA update algorithms. The approach where the butterfly individual learns from the optimal individual improves the algorithm’s exploration and exploitation capabilities. Reinforcement learning is performed once each for both local and global searches. Following constant iteration, the algorithm uses the methods of species migration and mutation to improve the performance of rapid convergence when it reaches a local extremum.

### 3.1. Move Formulation Incorporating Gaussian Mutation

The global update formula of the BOA can be expressed as follows:

(10) $$x_{i}^{t+1}=x_{i}^{t}\times \beta +(r^{2}\times g^{*}-x_{k}^{t})\times f_{i}$$

The local update formula is as follows:

(11) $$x_{i}^{t+1}=x_{i}^{t}+(r^{2}\times x_{j}^{t}-x_{k}^{t}\times \beta )\times f_{i}$$

In the above two equations, β is normrnd(0,δ(t)), that is, a random number with mean 0 and variance δ(t).

### 3.2. Update Strategy of Reinforcement Learning

**Figure 2:**
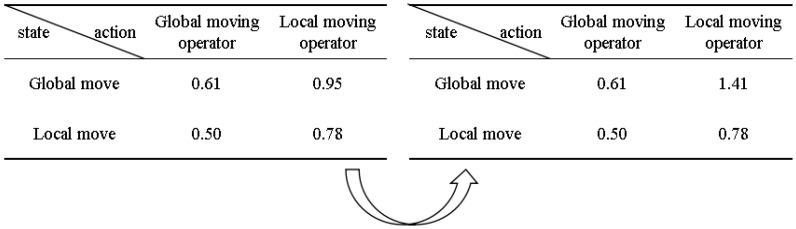
Examples of reinforcement learning Q-tables.

Combining the BOA and Q-learning algorithm, the QLBOA is proposed. The Q-learning algorithm replaces the control parameters of the BOA, that is, p, a, and c, with state–action pairs to indicate the selection of Q-values. The BOA has two stages—global search and local search—and the parameters of the algorithm determine which stage is carried out. The Q-table of the QLBOA is constructed as a 2 × 2 matrix, where the rows represent the states (*s_t_*) and the columns represent the actions for each state (*a_t_*), as shown in Figure 2.

The behavior of the Q-learner and the state to be learned are randomly chosen at the beginning of the search. The actions are determined and the state is updated according to the Q-table, which means that BOA search operators are selected based on past performance. Figure 2 shows the Q-table mapping of the QLBOA along with a numerical illustration. *r* = 1, *a_t_* = 0.69, *γ* = 0.80, and the current state–action pair is the *s_t_* global search operator, and *a_t_* is the local search machine operator. With $Q\left(s_{t},a_{t}\right)=0.95$, we update the new value of a in the Q-table according to the following equation:

(12) $$Q_{\left(t+1\right)}(s_{t},a_{t})=0.95+0.69[1+0.8\times \max(0.50,0.78)-0.95]=1.41$$

### 3.3. Migration and Mutation Mechanisms

**Figure 3:**
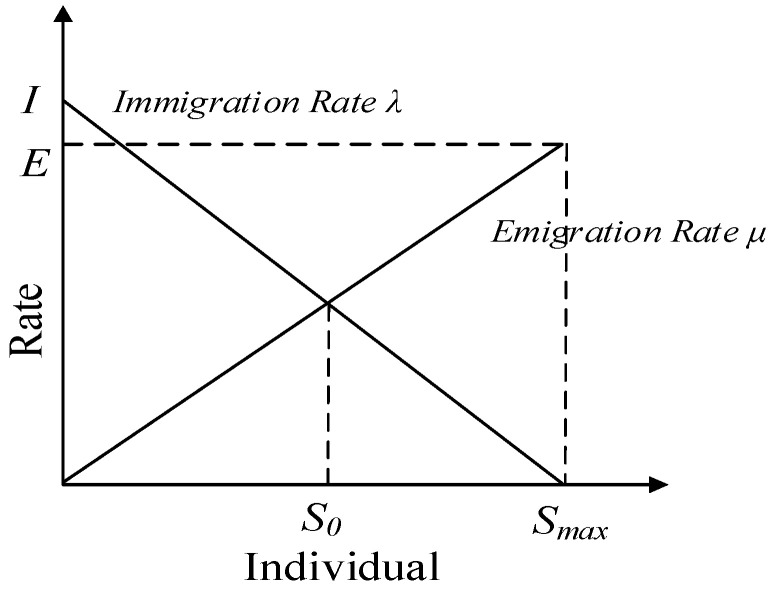
Change in mobility.

Inspired by the relationship between species migration and mutation, butterfly populations can evolve to a stable state without considering different species. However, when the number of predators or food changes, butterfly populations will also adjust in time by migrating or undergoing genetic changes to adapt to environmental changes. As shown in Figure 3, the maximum migration rate is *I* when no species are present in the habitat. As the number of species increases, the habitat becomes occupied, the chance of the successful survival of the migrants decreases, and the rate of migration decreases. The immigration rate becomes zero at $S_{\max}$. When the habitat contains no species, the emigration rate is zero. As the number of species increases, the habitat is crowded and more species can escape from the habitat; therefore, the emigration rate increases. The maximum value of the emigration rate is *E*. When the space contains species with equilibrium numbers, the two rates are equal at $S_{0}$.

The equations of migration rate *µ_k_* and migration rate λ*_k_* for *k* species are as follows:

(13) $$\mu_{k}=\frac{E\times n}{N}$$

(14) $$\lambda_{k}=I\times \frac{1-n}{N}$$

where *n* is the current number of butterflies, *N* is the maximum number of butterflies allowed, *E* is the maximum emigration rate, and *I* represents the maximum immigration rate.

The mutation mechanism improves the mining ability of the algorithm and maintains the diversity of the population as much as possible. The definition of this component is as follows:

(15) $$m_{n}=M\times \left(1-\frac{p_{n}}{p_{\max}}\right)$$

where *M* is the defined mutation, and *p_n_* is the mutation probability of the *n*th butterfly.

**Figure 4:**
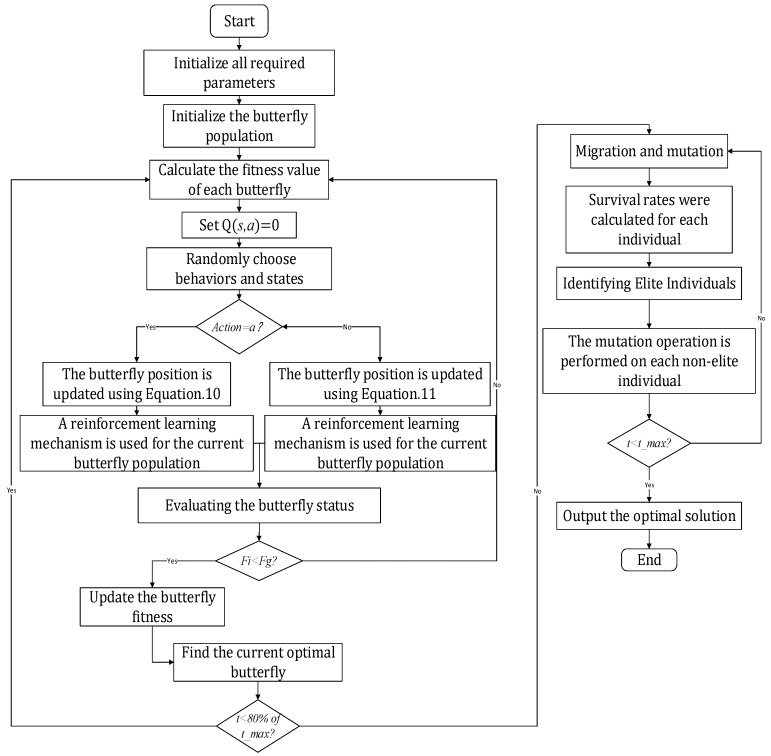
The flowchart of the QLBOA.

After calculating the fitness of each butterfly, the emigration rate, the immigration rate, and the mutation rate are updated. Non-elite individuals migrate and mutate according to these rates. The predefined best individual is saved as the elite of the next generation. Elitism prevents the best solution from being destroyed by migration. Figure 4 presents the proposed Algorithm 2 flow diagram.
**Algorithm 2** QLBOA
Generate the initial population of N butterflies.
Calculate the fitness of each search agent.
Sort the fitness and choose the best solution.
**While** t < 80% of the maximum number of iterations, **do**
      **For** each butterfly in the population, do
                      Calculate fragrance                      set Q(*s_t_*, *a_t_*) = 0      **End for**
      **For** each butterfly in the population, **do**
                Select action and state randomly.
                Select the best action at from the Q-table.
                **If** action == global search mechanism. then
                      Update position using Equation (6)
                **Else**
                      Update position using Equation (7)
                **End if**
                Evaluate the butterfly individual and update
            **End for**
**End while**
**While** 80% of the maximum number of iterations <= t < maximum number of iterations, **do**
            **For** each butterfly in the population, **do**
                  Calculate the fitness value and choose the elites.
                  Perform migration and mutation operations.
            **End for**
Calculate the fitness of each search agent.
Sort the fitness and choose the best solution.
**End while**
Output the best solution.


## 4. Simulation Experiments

### 4.1. Experimental Setup

The QLBOA’s efficacy was initially evaluated using eighteen complex functions in high dimensions. Table 1 details the parameters included in the comparison algorithms. In Table 2, the test functions are displayed. The suggested algorithm is contrasted with five traditional metaheuristics, namely, PSO, GA, ABC, DE, and BOA, and three BOA variants: IBOA [33], CBOA [34], and HPSOBOA [34].

The Table 3 CEC2022 optimization function test set has a total of 12 single-objective test functions with boundary constraints [35]. These are unimodal functions (F1), multimodal functions (F2–F5), hybrid functions (F6–F8), and combined functions (F9–F12). The test dimensions are 2, 10, and 20 dimensions, the same as the CEC2020 optimization function test set. All the test functions solve minimization problems. The CEC2022 optimization function test set is one of the latest test sets at present. The results are categorized using seven well-known algorithms: Differential Evolution (DE), Particle Swarm Optimization (PSO), Ant Colony Optimization (ACO), Artificial Bee Colony Algorithm (ABC), Sine Cosine Algorithm (SCA), Seagull Optimization Algorithm (SOA), and BOA.

Utilizing MATLAB R2019 (b), the method was applied. Experiments were carried out on a Windows 10 PC with an Intel (R) Core (TM) i5 CPU clocked at 3.30 GHz. There were exactly 30 butterflies in the population. Every technique employed in this study was run 30 times on its own, for a total of 500 iterations across all methods. To account for contingencies, all algorithms were run 30 times separately on each function, and the average minimal error (mean error) and standard deviation of the 30 runs were recorded. The first measures the algorithm’s convergence accuracy, while the second reflects its stability.

### 4.2. Analysis and Discussion of 18 Benchmark Functions’ Outcomes

This section compares the QLBOA on 18 benchmark functions with three BOA versions and five traditional metaheuristics. The algorithm’s stability is represented by the standard deviation, while its optimization accuracy is represented by the mean. The original table was too broad; therefore, the pertinent findings are displayed in two separate tables. Table 4 reveals what happened when the recommended strategy was compared to the other six algorithms. The results obtained using the QLBOA are the optima on most functions, with the exception of the cases on F7, F8, F17, and F18. Even more pleasantly, the QLBOA hits the optimal theoretical values on F1–F3 and F9–F15. The table’s findings demonstrate that, in terms of accuracy and optimization power, the enhanced algorithm performs better than the original BOA. The comparison findings between the three versions are shown in Table 5. It mostly illustrates the algorithm’s capacity for development with regard to unimodal functions. The multimodal functions are suitable for testing the local and global search capabilities. The convergence accuracy of the QLBOA on F7, F8, and F17 is lower than that on ABC, PSO, and GA, respectively. The convergence accuracy of the QLBOA on the remaining 15 functions is excellent, which shows that the adaptive Gaussian variation strategy can effectively improve the local development ability and improve the convergence accuracy of the algorithm.

**Figure 5:**
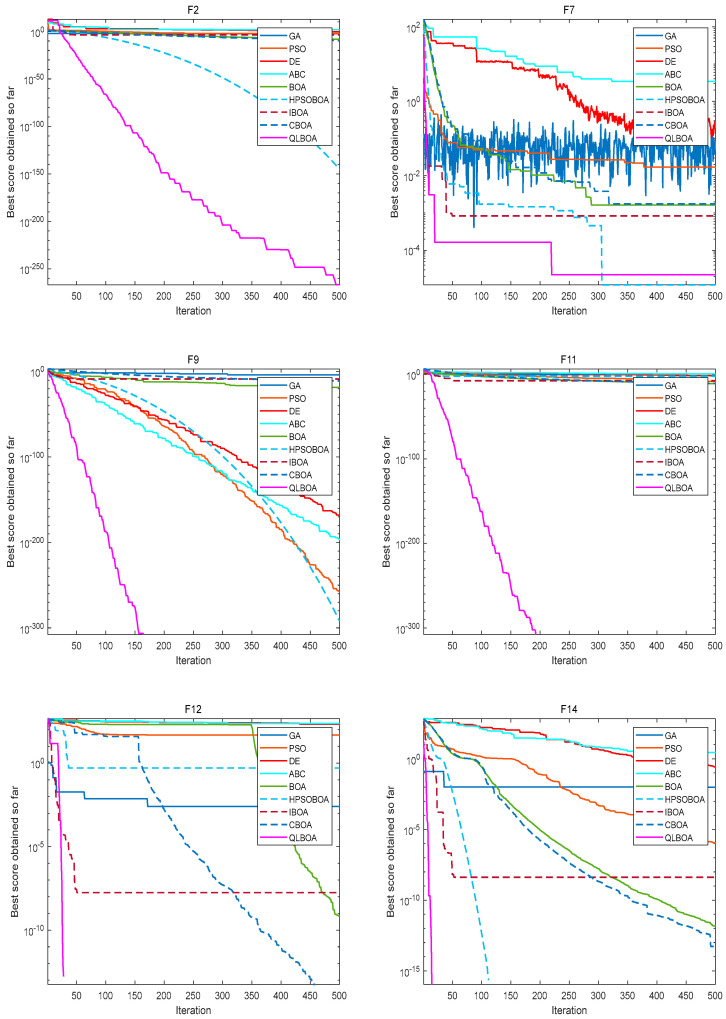
Comparison of convergence curves for *F*2, *F*7, *F*9, *F*11, *F*12, and *F*14.

**Figure 6:**
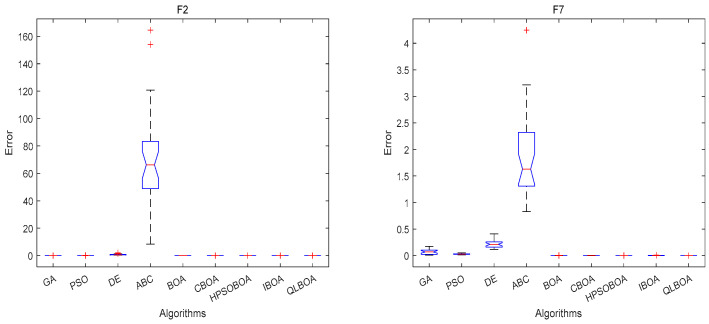

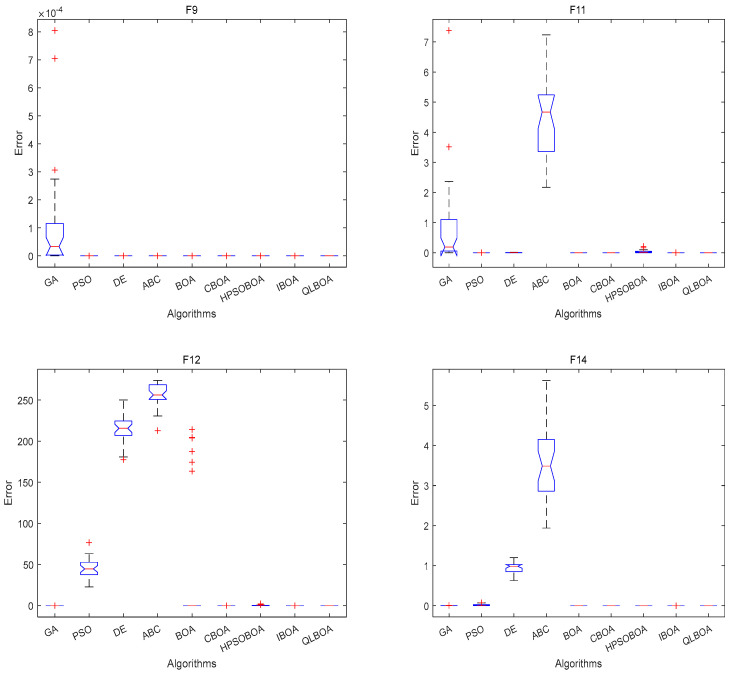
Error comparison for *F*2, *F*7, *F*9, *F*11, *F*12, and *F*14.

Figure 5 plots the convergence curves of different algorithms on nine benchmark functions, including F2, F7, F9, F11, F12, and F14. In general, the convergence of the QLBOA demonstrates that its convergence speed is faster and that its convergence accuracy is higher—even with the same iteration. The QLBOA technique converges swiftly in 20–30 generations and delivers the theoretical best value for the multimodal F12. Even though the QLBOA falls into the local optimum on F18 rather than the ideal value, it nevertheless exhibits a comparatively fast convergence rate. Figure 6 demonstrates that the QLBOA has the best stability, the smallest error, and the optimal difference when it comes to addressing the benchmark function problem.

Additional statistical work has been performed to support the QLBOA, including the Wilcoxon rank-sum test. Table 6 shows that the majority of the *p*-values are significantly less than 0.05, suggesting that the QLBOA and the other six algorithms differ significantly from one another.

### 4.3. Analysis and Discussion of CEC2022 Outcomes

CEC2022 was selected for the comparative test experiment in this section. D is the dimension size; therefore, D = 10. Table 7 displays the results of CEC2022 from the experiments. It can jump out of local optima in larger dimensions and has a good global search capacity. The mixed functions have a lot of extreme points, most of which indicate how effectively the algorithm can balance the stages of development and search. It is evident that most QLBOA results are closer to the optimal value and more accurate. The QLBOA offers great convergence speed and accuracy on the unimodal function *F*1, while other methods have poor convergence speed and low accuracy. None of the comparison algorithms were able to determine the exact solution on *F*6 except for the QLBOA. The QLBOA can easily enter the local optimum value on *F*9 and *F*11, much like other algorithms can, but in the middle and late iterations, it can successfully exit the local optimum. This is mostly due to the population’s implementation of the adaptive Gaussian mutation strategy and migration and mutation following each generation of evolution, which can enhance population quality and hasten population convergence.

**Figure 7:**
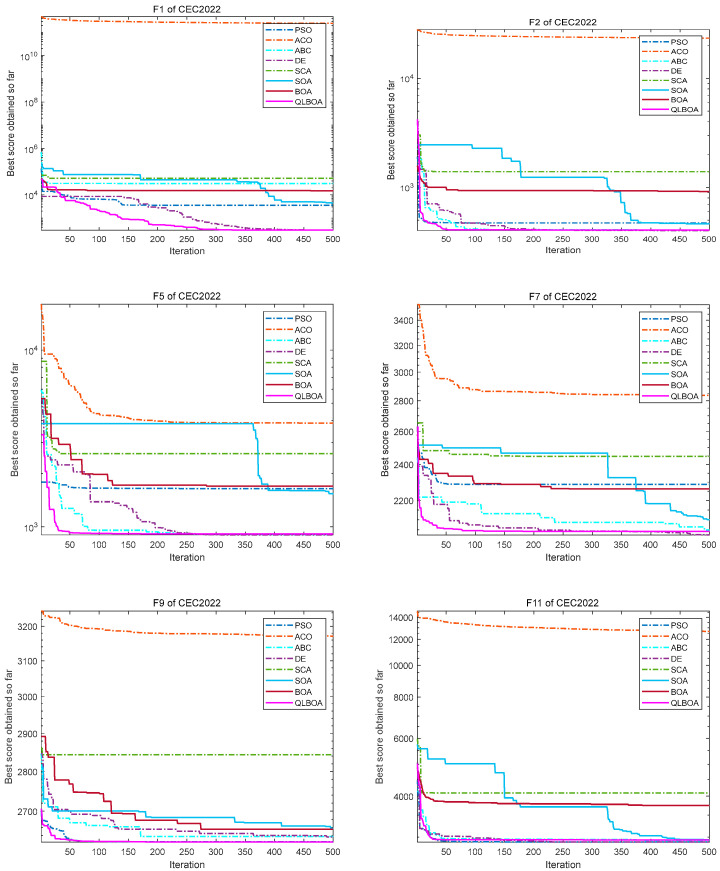
Comparison of convergence curves for *F*1, *F*2, *F*5, *F*7, *F*9, and *F*11 of CEC2022.

**Figure 8:**
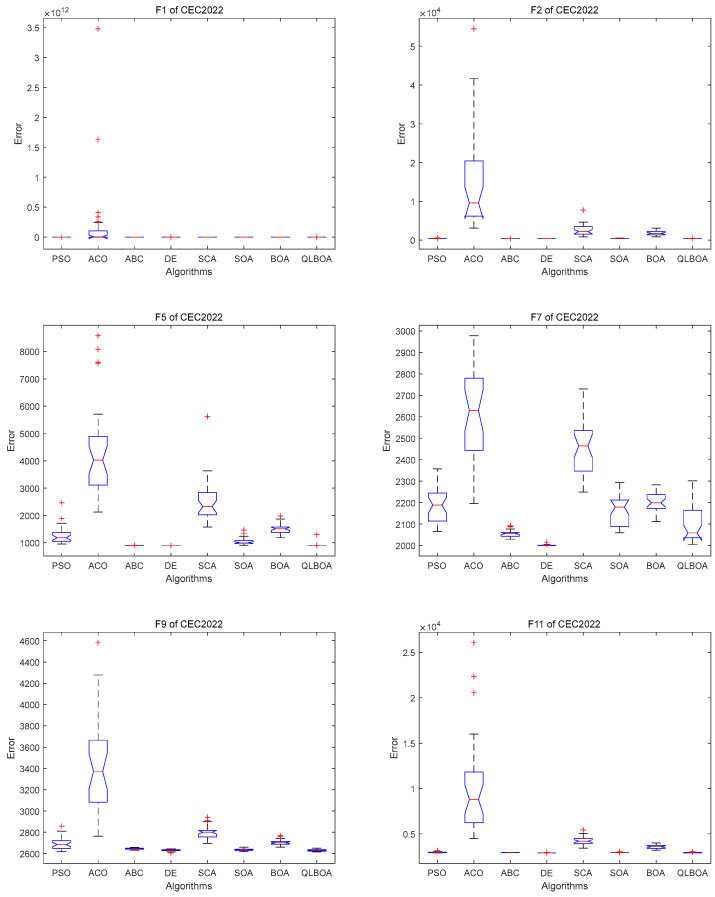
Error comparison for *F*1, *F*2, *F*5, *F*7, *F*9, and *F*11 of CEC2022.

The information in the table shows that the QLBOA has certain benefits when it comes to solving hybrid functions. The optimization capabilities of the comparison algorithms are displayed individually in Figure 7. The comparison algorithms’ inaccuracy is displayed in Figure 8. It is easier to compare each method’s performance. It shows that the QLBOA and DE perform better and are more stable than the other algorithms. The comparative experiment conducted on the CEC2022 suite has demonstrated that the QLBOA offers outstanding flexibility and global search capabilities. In addition to improving the individual quality of the population, the migration and variation strategy can further expand the optimization range of the population, maintain the diversity of the population, and help the algorithm to escape local optima and find other areas where there may be excellent solutions. The reinforcement learning mechanism can maintain the balance between the exploration and development capabilities of the algorithm and improve the optimization performance of the QLBOA. In addition, the population is able to broaden its search area, effectively exit the local optimum in the local scope, and assist in locating the region where the optimal solution is most likely to exist and converge swiftly. The QLBOA is an advanced algorithm that should be adopted because it has faster convergence speed, higher optimization accuracy, and more stability than the other nine comparison algorithms on the chosen CEC2022 suite, according to an analysis of the convergence curve and the results of the aforementioned numerical experiments. A comparison of the Wilcoxon rank-sum test findings is displayed in Table 8.

Additional statistical work was conducted to support the QLBOA, including the Wilcoxon rank-sum test. Table 8 shows that the optimization performance is compare with other methods and that there is little discernible difference between the proposed algorithm and DE and ABC for *F*3, *F*7, *F*9, and *F*11. The majority of the p values are significantly smaller than 0.05, suggesting that the QLBOA method and the other six algorithms differ from one another.

### 4.4. Computational Complexity of BOA and QLBOA

The population’s initialization is one of the key processes in the BOA: $O\left(N\right)$. The optimal population is selected by classifying the starting population’s fitness: $O\left(N^{2}\right)$. Updating the population occurs throughout the exploration phase: $O\left(N\right)$. During the exploration phase, the updating population is $O\left(2N\right)$. The fitness value is calculated, and the best option is selected: $O{\left(N\right)}^{2}$. The temporal complexity of the BOA is $2\times O\left(N\right)+O\left(N^{2}\right)+O\left(2N\right)+O{\left(N\right)}^{2}$.

In the QLBOA, the population’s initialization is determined by its size: $O\left(N\right)$. The starting population’s fitness is determined: $O\left(N\right)$. The reinforcement learning strategy is applied to the whole population: $O\left(2N\right)$. Updating of the population occurs throughout the exploration phase: $O\left(N+2N^{2}\right)$. The best option is selected after classifying the population: $O\left(N^{2}\right)$. The fitness value is calculated, and the best option is selected: $O\left(N+N^{2}\right)$. The QLBOA’s temporal complexity is $O\left(N\right)+O\left(N\right)+O\left(N^{2}\right)+O\left(2N\right)+O\left(N+2N^{2}\right)+O\left(N+N^{2}\right)$.

## 5. The QLBOA Solves the Green Vehicle Routing Problem Considering Customer Preferences

### 5.1. Description of the Vehicle Routing Problem

**Figure 9:**
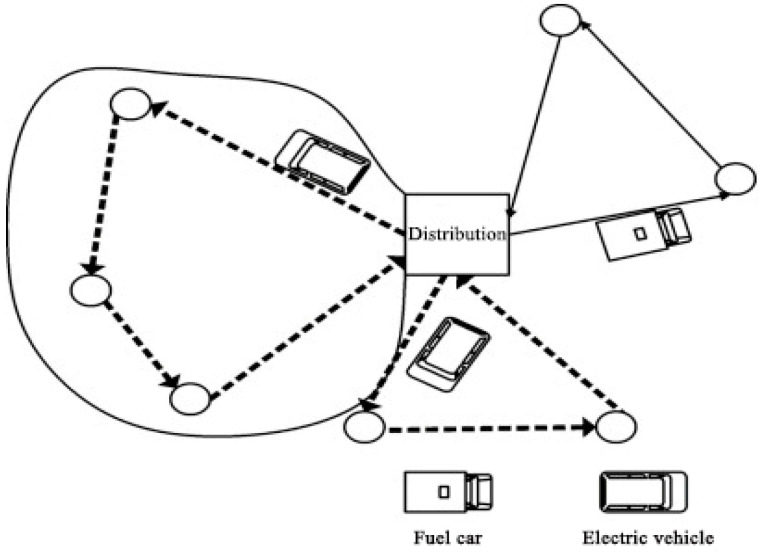
Green vehicle routing problem.

This section focuses on freight distribution and introduces the QLBOA strategy to address the green vehicle routing problem with time windows. It combines the time-dependent speed and piecewise penalty cost for early and late deliveries. The goal is to establish a cooperative relationship that allows idle vehicles to serve customers at any facility, transforming soft time windows into piecewise penalty costs based on arrival time and customer characteristics. Customers are categorized as loyal or general, with delayed delivery to loyal customers assumed to have a greater negative impact on firm stability than other companies. The operating costs include transportation, fuel, and penalty costs. Transportation resources in the same warehouse are shared to improve resource management, optimize routes, and reduce environmental impacts. A mixed-integer mathematical model is developed for carbon emission and cost minimization considering the time dependence of vehicle speed and piecewise penalty costs. Two types of penalties are used: a constant waiting penalty for early arrivals that do not affect on-time delivery, and a variable delay penalty for late arrivals (Figure 9).

The location distribution of customers is different, and the service time window is also different. After a customer places an order, the historical order demand is known, but the actual customer demand is unknown. After the vehicle loads enough goods, it will deliver the order to each customer according to the established route. The vehicle can arrive at the customer point in advance but cannot be late. The service must be carried out within the time window.

The whole distribution process should minimize the total distribution cost of the distribution center. The research problem model in this section needs to match the real situation. In order to better present the model, the following assumptions are put forward:

(1) Customer requirements are independent of each other, and they will be updated only after the vehicle arrives at the customer point;
(2) Vehicles depart from and return to the distribution center;
(3) Vehicle use has a transportation cost, fuel cost, and penalty cost;
(4) The quantity of goods delivered can meet the predicted demand and actual demand of customers.

### 5.2. Problem Model

Model symbols and parameter settings see Table 9.

#### 5.2.1. Soft Time Window

**Figure 10:**
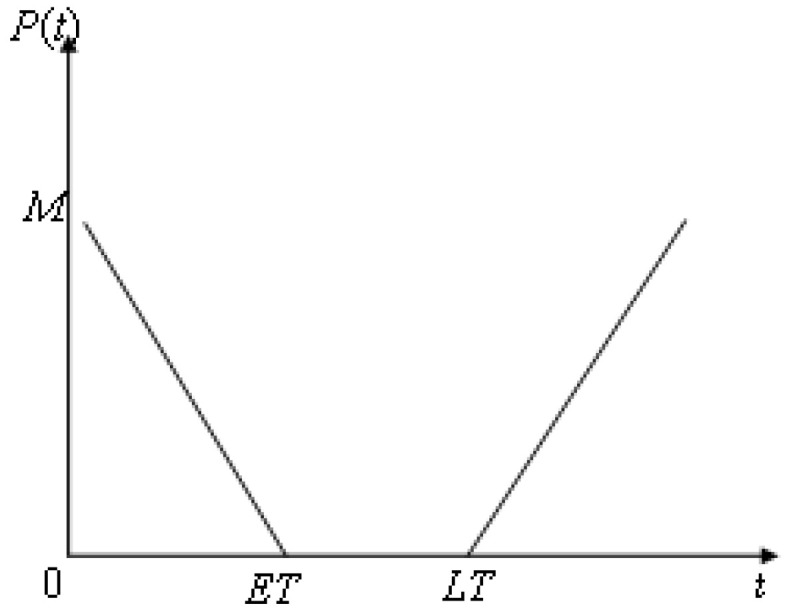
The relationship between punishment cost and time.

After setting the soft time window, the vehicle needs to provide service within the agreed time. If it does not arrive within the specified time, it can still provide service to the customer, but it will be punished according to the degree of early arrival or late arrival, and a certain penalty cost needs to be paid [44]. The relation between the penalty cost and time is shown in Figure 10.

#### 5.2.2. Vehicle Speed

**Figure 11:**
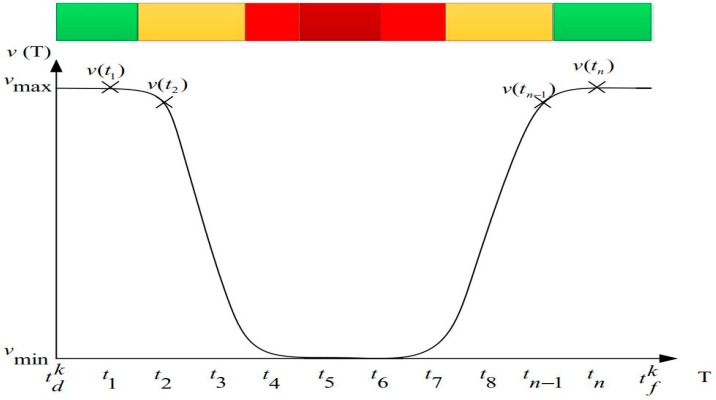
The relationship between vehicle speed and time.

As shown in Figure 11, a normal distribution of traffic density is assumed, taking into account the transition from a condition of free flow to one of extreme congestion. Since severe congestion may cause a full stop, each vehicle’s speed is defined to follow a normal distribution, with *v_min_* = 0 m/s; the maximum speed permitted by traffic regulations is *v_max_*. A vehicle is not allowed to travel on a specific road (*i*, *j*) in the same amount of time under particular traffic circumstances. Consequently, the total fuel consumption on (*i*, *j*) is determined by adding the fuel consumption at each time period. Within the *t_n_* time interval [*t_n−_*_1_, *t_n_*], the vehicle speed is represented by the following equation:

(16) $$\upsilon_{ijk}^{t_{n}}=\frac{\upsilon \left(t_{n}\right)+\upsilon \left(t_{n-1}\right)}{2}$$

#### 5.2.3. Fuel Consumption

Vehicle carbon emissions are calculated, and the fuel consumption rate of vehicle *k* on (*i*, *j*) can be regarded as the carbon emission rate of the vehicle. Assuming that *x* kg carbon is emitted from no gasoline, the fuel consumption rate of vehicle *k* on the road section (*i*, *j*) is

(17) $$f_{ijk}=\frac{c_{ijk}}{x}$$

where *c* represents the carbon emission rate (kg/km), and the expression is as follows:

(18) $$c_{ijk}=\frac{\varphi_{\upsilon }\psi }{1000}$$

The carbon emission rate of vehicle *k* in g/km is

(19) $$\varphi_{\upsilon }=\omega_{0}+\omega_{1}\upsilon +\omega_{2}\upsilon^{2}+\omega_{3}\upsilon^{3}+\frac{\omega_{4}}{\upsilon }+\frac{\omega_{5}}{\upsilon^{2}}+\frac{\omega_{6}}{\upsilon^{3}}$$

Here, *φ_v_* is the carbon emission rate at velocity *v*, and *ω*_0_ to *ω*_6_ are constants. The carbon emission rate load correction factor is

(20) $$\psi =\chi_{0}+\chi_{1}\gamma +\chi_{2}\gamma^{2}+\chi_{3}\gamma^{3}+\chi_{4}\upsilon +\chi_{5}\upsilon^{2}+\chi_{6}\upsilon^{3}+\frac{\chi_{7}}{\upsilon }$$

Here, *γ* represents the ratio of the actual load and capacity of the vehicle on the road section (*i*, *j*), and *χ*_0_ to *χ*_7_ are constants.

#### 5.2.4. Penalty Costs

Delivery vehicles arriving from I before the earliest time restriction may cause needless waiting before offloading, which might potentially affect other customers’ punctual deliveries, resulting in delays and lower customer satisfaction. If service is started at node j within the permitted time frames, there is no penalty. Thus, the distribution company can optimize service dependability and customer happiness by following the client’s time interval. By modifying vehicle departure times, a vehicle routing solution that satisfies these requirements can be used as a model for efficiently cutting operating expenses and preventing traffic jams. A lateness penalty factor *δ_lj_* is applied in the event that the delivery vehicle comes over the closest time restriction.

(21) $$\mu_{j}=\left\{\begin{array}{ll} \delta_{e}[ET_{j}-t_{ijk}-t_{i}] & t_{ij}+t_{i}<ET_{j}\\ 0 & ET_{j}<t_{ijk}+t_{ik}<LT_{j}\\ \delta_{lj}[t_{ijk}+t_{ik}-LT_{j}] & t_{ijk}+t_{i}>LT_{j} \end{array}\right.$$

### 5.3. Objective Function


(22) $$\begin{array}{l} \min{\;F}_{1}\left(x,y,z,\lambda_{1},\lambda_{2},\lambda_{3}\right)=\left(1-\lambda_{1}\right)\cdot \left(C_{k}\sum_{i\in N}\sum_{j\in N}\sum_{k\in K}x_{ijk}d_{ij}+C_{v}\sum_{k\in K}z_{k}\right)\\ +\left(1-\lambda_{2}\right)\cdot \left(C_{f}\sum_{i\in N}\sum_{j\in N}\sum_{k\in K}x_{ijk}f_{ijk}d_{ij}+C_{e}\sum_{i\in N}\sum_{j\in N}\sum_{k\in K}x_{ijk}e_{ijk}d_{ij}\right)\\ +\left(1-\lambda_{3}\right)\cdot \sum_{j\in N}\sum_{k\in K}\mu_{j}y_{jk}z_{k} \end{array}$$

(23) $$s.t.\;\;\;\;\;\mathrm{Pr}\left\{\left.\sum_{i\in N^{\prime }}q_{i}y_{ij}\leq Q\right\}\right.\geq \varepsilon ,\forall k\in K$$

(24) $$\sum_{j\in N^{\prime }}x_{0jk}=\sum_{j\in N^{\prime }}x_{j0k},\forall k\in K$$

(25) $$\sum_{j\in N^{\prime }}x_{0jk}\leq 1,\forall k\in K$$

(26) $$\sum_{j\in N^{\prime }}x_{j0k}\leq 1,\forall k\in K$$

(27) $$\sum_{k\in K}\sum_{i\in N}x_{ilk}=\sum_{k\in K}\sum_{j\in N}x_{ljk}\leq K,\forall l\in N^{\prime }$$

(28) $$\sum_{i\in N\prime }\sum_{k\in K}q_{0jk}^{+}x_{0jk}\geq \sum_{i\in N\prime }{\hat{q}}_{i}$$

(29) $$\sum_{k\in K}y_{ik}=1,\forall i\in N$$

(30) $$\sum_{j\in N}x_{ijk}=y_{ik},\forall i\in N^{\prime },\;\forall k\in K$$

(31) $$\sum_{i\in N}y_{ik}=z_{k},\forall k\in K$$

(32) $$t_{ijk}=d_{ij}/\upsilon_{k},\;\forall k\in K,\forall i,j\in N$$

(33) $$x_{ijk}\in \left\{\left.0,1\right\}\right.,\;\forall i,j\in N,i\neq j,\forall k\in K$$

(34) $$x_{ijk}\in \left\{\left.0,1\right\}\right.,\;\forall i,j\in N,i\neq j,\forall k\in K$$

Equation (22) represents the optimization of the vehicle transportation cost, fuel cost, and penalty cost with weighting factors *λ*_1_, *λ*_2_, *λ*_3_ ∈ [0, 1].

Equation (23) ensures that the probability of customer demand being less than the load in the vehicle distribution path is greater than a specified confidence level.

Equations (24)–(26) represent the departure and return of vehicles from/to the distribution center.

Equation (27) indicates that the number of vehicles passing through the distribution center or a customer point is consistent and does not exceed a maximum limit.

Equation (28) ensures the satisfaction of all customer requirements.

Equation (29) ensures that each customer is served by one vehicle only.

Equations (30) and (31) represent relationships between 0 and 1 variables.

Equation (32) represents travel time for vehicle *k* from customer point *i* to *j*.

Equations (33) and (34) represent 0–1 decision variables.

### 5.4. The Flow of the QLBOA to Solve the Problem

**Figure 12:**
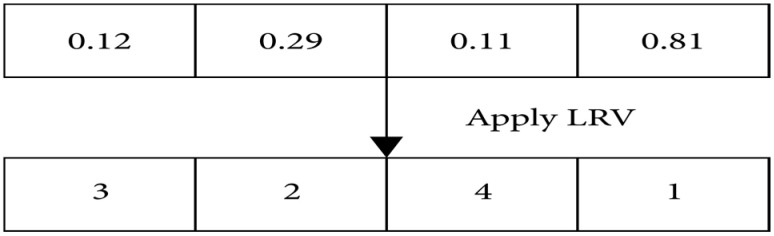
LRV application process.

The upper and lower boundaries of *ϕ* determine where the initial search agent is located in this subsection. The placement of the QLBOA agent is determined by setting upper and lower limit values. During this stage, the search agent’s position is confirmed and its duplicate number is checked. Guidelines for converting LRVS from search agent locations (continuous data) to trip sequences (discrete values) are obtained in [5,45]. In combinatorial problems, LRV is a frequently used technique for converting continuous to discrete values. As seen in Figure 12, each search agent’s position data are sorted in this phase from the maximum to the minimum. The search agent location cannot be applied when visiting the sequence/route of vehicles at the same two locations.

### 5.5. Datasets and Parameter Settings

**Figure 13:**
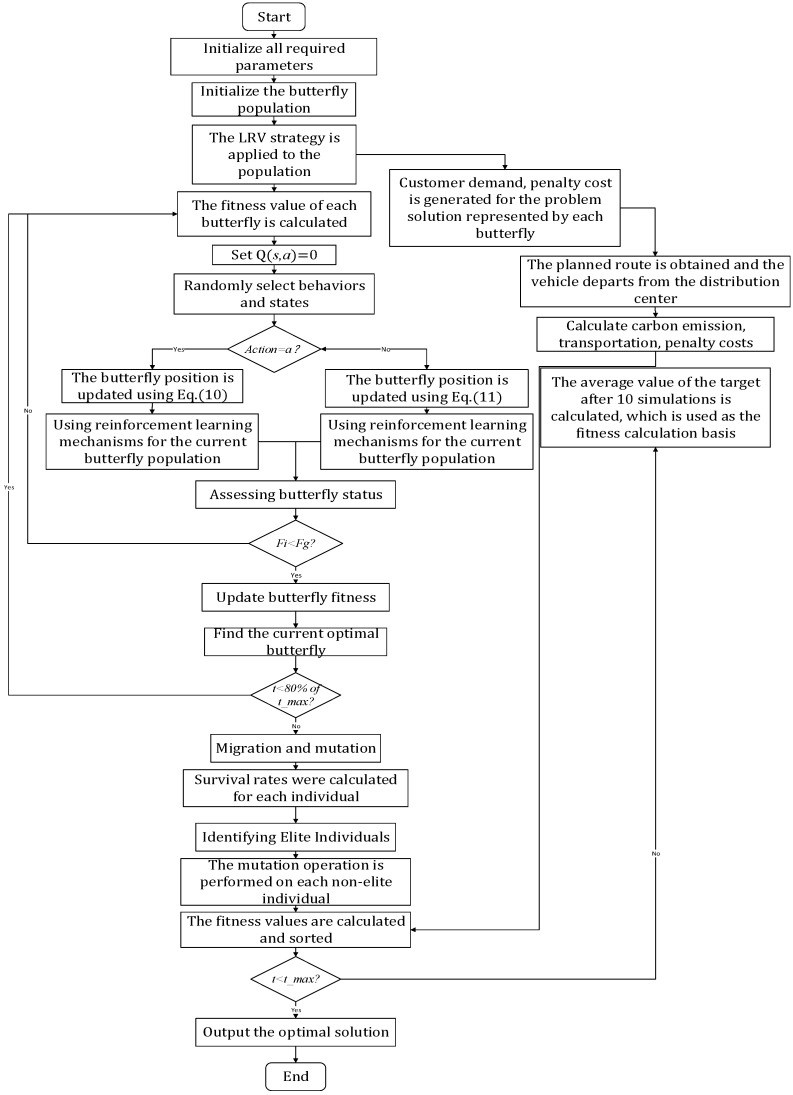
QLBOA solution process for green vehicle routing problems with time windows.

The QLBOA was used to solve the green vehicle routing problem with time windows to minimize carbon emissions and operating costs, and the simulation experiment was carried out by using the examples in the Solomon VRPTW standard test library [46]. The calculation examples in the standard test library include six data types: random distributions (R1, R2), clustering distributions (C1, C2), and random and clustering mixture distributions (RC1, RC2). In this section, two datasets are randomly selected from various data types to test the influence of the algorithm. Table 10 shows the various parameter settings for the problem. The QLBOA process for solving the green vehicle routing problem with time windows is shown in Figure 13.

### 5.6. Response Analysis

#### 5.6.1. Analysis of the Influence of Decision Makers’ Subjective Preferences on Goals

**Figure 14:**
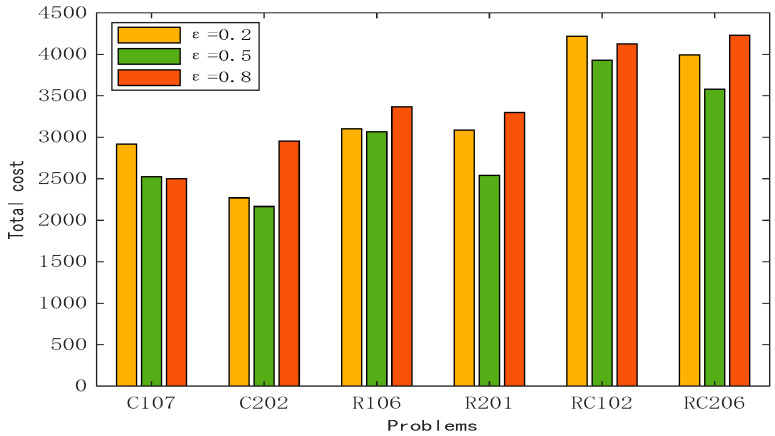
Analysis of the impact of decision makers’ subjective preferences on total costs.

The subjective preferences of decision makers determine the utilization rate of vehicles, and the subjective preference value is usually between 0 and 1. With the increase in the subjective preference value, the customer’s requirements for the quality of the service received will be higher and higher; otherwise, more attention will be paid to the utilization rate of vehicles so as to reduce the use cost of vehicles. This subsection keeps the other parameters unchanged and analyzes the changes in the transportation cost, fuel cost, and penalty cost when *ε* changes to three values: 0.2, 0.6, and 0.8. Meanwhile, the number of vehicles used is counted. It can be seen in Table 11 that when *ε* is small, although the number of vehicles used in the planned route is small, the fuel cost and vehicle use cost are high, and the penalty cost in the transportation process is high. When *ε* is 0.6, the cost of most routes reaches the minimum. When *ε* is 0.8, the subjective preference of decision makers increases, the service process gives priority to customer satisfaction, and the penalty cost decreases, but the transportation cost and fuel cost recover, and the number of vehicles used reaches the highest value. As shown in Figure 14, the lowest total cost is shown with a green cylinder, and as the subjective preference increases, the total service cost shows a trend of first decreasing and then increasing. Based on the analysis of the graph, *ε* is set to 0.6. The figure shows that the total cost of the example of type C is relatively low, while R and RC are relatively high. This is due to the fact that the customer locations in the example of type C are clustered and concentrated, and the vehicle does not need to cross long distances to complete the service of multiple customers in a small range. The increase in vehicles in the service process will increase the transportation cost and fuel cost, and the total cost will also increase.

#### 5.6.2. Analysis of the Impact of Weight Factors on the Target

**Figure 15:**
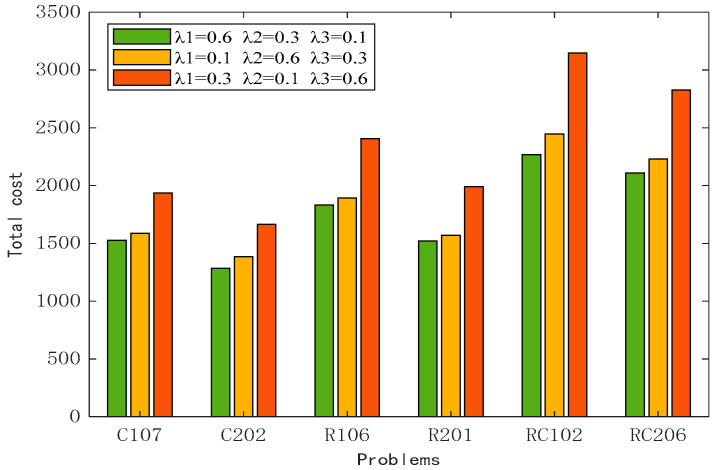
Analysis of the impact of weight factors on the total cost.

**Figure 16:**
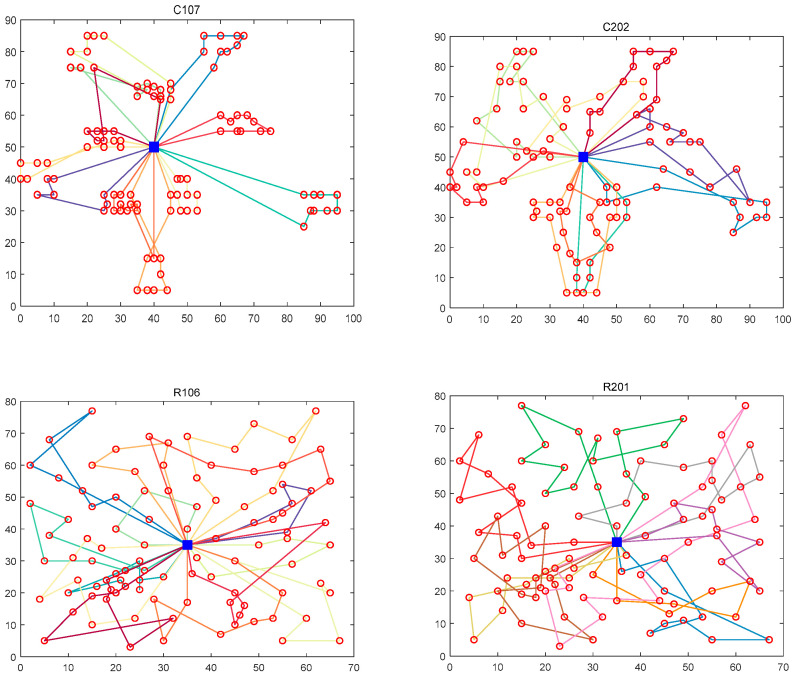

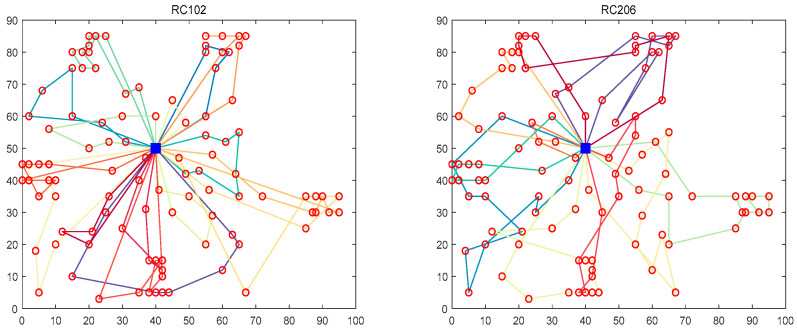
QLBOA’s path to solving different problem sets.

When *λ*_1_ = 0.6, *λ*_2_ = 0.3, and *λ*_3_ = 0.1, the model focuses on transportation costs, followed by fuel costs, and does not fully consider customer satisfaction. When *λ*_1_ = 0.1, *λ*_2_ = 0.6, and *λ*_3_ = 0.3, the model focuses on fuel consumption and carbon emissions in the service process and considers environmental protection an important task. When *λ*_1_ = 0.3, *λ*_2_ = 0.1, and *λ*_3_ = 0.6, the service focuses on customer demand and strives to minimize the penalty cost in the service process. The results of various types of costs for the above three cases are shown in Table 12. Taking transportation cost as an example, the cost obtained by adopting the first scheme is the lowest in all kinds of examples, and the difference between the first scheme and the highest cost is at most 12.53%. For the fuel cost, the cost of adopting the second scheme is the lowest, and the difference from the highest cost is up to 11.24%. The service penalty cost is lowest when customer requirements are focused on. The total generation cost for each case is shown in Figure 15. When customer satisfaction is not fully considered, the total cost can reach the lowest value. The reason is that the penalty factor in the calculation formula of the penalty cost is 100, and the opening-to-ending span of the time window is small, so the penalty cost is far less than the transportation cost and the fuel cost. After adding the weight factor, the penalty cost has a smaller impact on the total cost. For the model proposed in this section, it is necessary to comprehensively consider the service path, not only considering the economic cost and carbon emissions but also based on the brand image to improve customer satisfaction. Therefore, the weight factor is introduced. Based on the above analysis, the set of weight values with the lowest total cost is selected as the final plan, and the path is shown in Figure 16.

#### 5.6.3. Comparison with Other Algorithms

**Figure 17:**
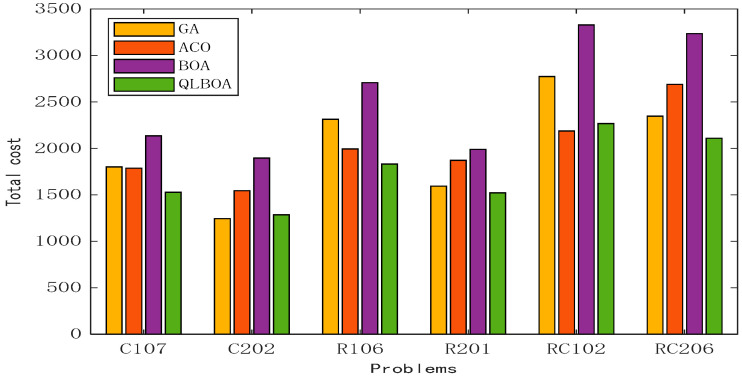
Comparison of total costs between QLBOA and three other algorithms.

Table 13 shows the comparison results between the QLBOA and the other three metaheuristics. The improved algorithm achieves small values for transportation cost, fuel cost, and penalty cost. Compared with the basic BOA, the effect of the QLBOA’s optimization objective is greatly improved. As shown in Figure 17, the average of the total cost is calculated after each algorithm is run 10 times. In examples C202 and RC102, the total generation cost of the QLBOA is greater than that of the GA and ACO, respectively, and in other examples, the QLBOA achieves the greatest effect.

## 6. Conclusions and Future Work

This work proposes the butterfly optimization algorithm with Q-learning (QLBOA). This paper is innovative in that it introduces a reinforcement learning mechanism, combines the butterfly optimization algorithm with dynamic Gaussian variation, integrates the butterfly renewal strategy with the strategy for species migration and mutation, and increases the diversity of the algorithm population. Premature convergence is successfully avoided, mutual benefits are realized, and the global optimal value is solved more quickly in the search space. It was tested by solving the CEC2022 test suite and eighteen single-objective benchmark functions, as well as a range of traditional metaheuristic algorithms. Lastly, the fuel consumption, carbon emissions, and penalty costs of vehicles in the process of delivering goods and serving customers were adopted as the optimization objectives, and the QLBOA was employed to solve the green vehicle routing problem, taking customer wants into consideration. An analysis was performed on the impact of decision makers’ subjective preferences and weight factors on various optimization costs as well as total costs. Carbon emissions rise in response to an increase in the penalty cost weight factor, which is counterproductive to environmental preservation. Ultimately, the results demonstrate that the QLBOA technique suggested in this study has certain advantages when compared with three heuristic optimization strategies. Consequently, the QLBOA exhibits considerable potential for use in the industrial control domain. Furthermore, the QLBOA can be expanded to address multi-objective scenarios in the resolution of progressively complex engineering optimization problems.

## Figures and Tables

**Table 1:** Parameter settings.

Algorithms	Name	Parameter Settings
PSO	Particle Swarm Optimization	*a* = 0.3, *b* = 1, *c* = 1
ABC	Artificial Bee Colony Algorithm	*m* = 0.2
ACO	Ant Colony Optimization	*c* = 10^−6^, *Q* = 20, *m* = 1,
GA	Genetic Algorithm	*Qc* = 1, *Qm* = 0.01
DE	Differential Evolution Algorithm	*q* = 0.2, *α*1 = 0.8, *α*2 = 0.2
SCA	Sine Cosine Algorithm	*α* = 2, c1 = *b* − t × (*b*/*T*),
SOA	Seagull Optimization Algorithm	*c* = 1
BOA	Butterfly Optimization Algorithm	*p* = 0.8, *α* = 0.1, *c =* 0.01
CBOA	Optimization Algorithm with Cubic Map	*a*_1_ = 0.1, *a*_2_ = 0.3, *c* = 0.01, *p* = 0.6, *m* = 0.315, *P* = 0.295
HPSOBOA	Hybrid PSO with BOA and Cubic Map	*a*_1_ = 0.1, *a*_2_ = 0.3, *c* = 0.01, *p* = 0.6, *x* = 0.315, *P* = 0.295, *c*1 = *c*2 = 0.5
IBOA	Improved BOA	*a* = 0.1, *c* = 0.01, *P* = 0.6, *x* = 0.33, *w* = 4
QLBOA	BOA with Q-learning	*p* = [0.1, 0.8], *α* = 0.1, *c =* 0.01, *m* = 0.1, *e* = 0.4

**Table 2:** Eighteen benchmark functions.

Type	No.	Functions	Search Ranges	*F*_min_
High-dimensional unimodal	*F*1	Schwefel’s Problem 1.2	[−100, 100]	0
	*F*2	Generalized Rosenbrock’s Function	[−10, 10]	0
	*F*3	Sphere Function	[−100, 100]	0
	*F*4	Schwefel’s Problem 2.21	[−100, 100]	0
	*F*5	Schwefel’s Problem 2.22	[−10, 10]	0
	*F*6	Sum-of-Different-Powers Function	[−100, 100]	0
	*F*7	Quartic Function, i.e., Noise	[−1.28, 1.28]	0
	*F*8	Bent Cigar Function	[−10, 10]	0
	*F*9	Step Function	[−100, 100]	0
	*F*10	Zakharov Function	[−5, 10]	0
	*F*11	Discus Function	[−5, 5]	0
High-dimensional multimodal	*F*12	Generalized Rastrigin’s Function	[−5.12, 5.12]	0
	*F*13	Ackley’s Function	[−32, 32]	0
	*F*14	Generalized Griewank’s Function	[−600, 600]	0
	*F*15	HappyCat Function	[−50, 50]	0
	*F*16	Lévy Function	[−10, 10]	0
	*F*17	Katsuura Function	[−50, 50]	0
	*F*18	HGBat Function	[−20, 20]	0

Note: x* stands for the global optima. *F* is the fitness value. D = 30.

**Table 3:** CEC2022 test suite.

Type	NO.	Functions	*F*_min_
Unimodal Functions	1	Shifted and full Rotated Zakharov Function	300
Multimodal Functions	2	Shifted and full Rotated Rosenbrock’s Function	400
	3	Shifted and full Rotated Rastrigin’s Function	600
	4	Shifted and full Rotated Non-Continuous Rastrigin’s Function	800
	5	Shifted and full Rotated Lévy Function	900
Hybrid Functions	6	Hybrid Function 1 (N = 3)	1800
	7	HF 2 (N = 6)	2000
	8	HF 3 (N = 5)	2200
Composition Functions	9	Composition Function 1 (N = 5)	2300
	10	CF 2 (N = 4)	2400
	11	CF 3 (N = 5)	2600
	12	CF 4 (N = 6)	2700

Search range: [−100, 100]^D^; “HF” means “Hybrid Function”, and “CF” means “Composition Function”.

**Table 4:** The results are compared with those of five other algorithms on benchmark functions.

Function		GA [36]	DE [37]	PSO [37]	ABC [38]	BOA [28]	QLBOA
*F*1	Mean	1.4181 × 10^+03^	3.8513 × 10^−03^	6.8100 × 10^−13^	2.6770 × 10^−16^	2.5100 × 10^−11^	**8.0212 × 10^−231^**
	Std	5.9444 × 10^+02^	1.0000 × 10^−02^	5.3000 × 10^−13^	6.4934 × 10^−17^	1.9300 × 10^−12^	**0.0000 × 10^+00^**
*F*2	Mean	2.4766 × 10^+01^	−2.0602 × 10^−00^	2.0892 × 10^−02^	3.1462 × 10^−09^	2.3900 × 10^−11^	**0.0000 × 10^+00^**
	Std	5.2444 × 10^+00^	9.2312 × 10^−08^	1.4800 × 10^−01^	5.3864 × 10^−09^	2.2800 × 10^−12^	**0.0000 × 10^+00^**
*F*3	Mean	2.2230 × 10^+04^	−1.0000 × 10^−00^	1.4184 × 10^−05^	9.3412 × 10^−10^	2.2400 × 10^−11^	**0.0000 × 10^+00^**
	Std	4.4852 × 10^+03^	3.1712 × 10^−06^	5.9800 × 10^+02^	8.9224 × 10^−03^	1.8800 × 10^−12^	**0.0000 × 10^+00^**
*F*4	Mean	5.1304 × 10^+01^	−2.8732 × 10^−00^	1.4184 × 10^−05^	5.9962 × 10^−10^	1.1900 × 10^−08^	**1.3380 × 10^−249^**
	Std	6.4693 × 10^+00^	1.5538 × 10^−12^	8.2700 × 10^−06^	2.3114 × 10^−12^	8.3500 × 10^−10^	**0.0000 × 10^+00^**
*F*5	Mean	6.9558 × 10^+03^	1.7400 × 10^−01^	3.5600 × 10^+02^	2.9732 × 10^−10^	2.8900 × 10^+01^	**4.7112 × 10^−02^**
	Std	9.7903 × 10^+03^	2.1200 × 10^−01^	2.1500 × 10^+03^	3.5514 × 10^+01^	9.5400 × 10^−02^	**5.2924 × 10^−02^**
*F*6	Mean	9.5971 × 10^+02^	−4.1413 × 10^−00^	4.0300 × 10^−02^	4.9872 × 10^−17^	5.1700 × 10^+00^	3.6750 × 10^−03^
	Std	2.5531 × 10^+02^	1.6542 × 10^−02^	3.9800 × 10^−01^	4.6481 × 10^−14^	6.3900 × 10^−01^	**3.3326 × 10^−03^**
*F*7	Mean	3.5458 × 10^−01^	1.1500 × 10^−00^	1.4082 × 10^−04^	**7.3670 × 10^−14^**	4.0300 × 10^−03^	1.1574 × 10^−04^
	Std	7.3510 × 10^−02^	0.2300 × 10^−00^	1.1400 × 10^−03^	**5.3882 × 10^−09^**	8.7000 × 10^−04^	1.1050 × 10^−04^
*F*8	Mean	2.8900 × 10^+01^	1.5000 × 10^−02^	**7.3800 × 10^−61^**	6.0962 × 10^−03^	6.5100 × 10^−17^	5.5810 × 10^−02^
	Std	2.5400 × 10^−02^	4.0414 × 10^−02^	**3.8102 × 10^−60^**	7.3131 × 10^−03^	1.3900 × 10^−16^	2.8200 × 10^−01^
*F*9	Mean	1.5721 × 10^+01^	2.0000 × 10^−08^	1.3712 × 10^−14^	1.8780 × 10^−14^	6.3300 × 10^−14^	**0.0000 × 10^+00^**
	Std	5.1484 × 10^+00^	5.3312 × 10^−08^	4.6430 × 10^−14^	2.4251 × 10^−13^	3.4000 × 10^−14^	**0.0000 × 10^+00^**
*F*10	Mean	1.4434 × 10^+01^	−1.8732 × 10^+02^	9.0222 × 10^−05^	6.5802 × 10^−05^	6.7200 × 10^−11^	**0.0000 × 10^+00^**
	Std	8.4536 × 10^−01^	3.3950 × 10^−04^	1.0544 × 10^−04^	1.4841 × 10^−05^	6.9000 × 10^−12^	**0.0000 × 10^+00^**
*F*11	Mean	1.5250 × 10^+01^	4.3300 × 10^−03^	9.0221 × 10^−05^	7.8280 × 10^−04^	6.7200 × 10^−11^	**0.0000 × 10^+00^**
	Std	7.3036 × 10^+00^	1.9000 × 10^−02^	1.0504 × 10^−04^	2.2000 × 10^−04^	6.9000 × 10^−12^	**0.0000 × 10^+00^**
*F*12	Mean	3.0789 × 10^+00^	3.1300 × 10^−03^	8.5598 × 10^−03^	3.2000 × 10^−08^	2.5100 × 10^+01^	**0.0000 × 10^+00^**
	Std	1.8282 × 10^+00^	9.5412 × 10^−03^	4.7900 × 10^−02^	2.2112 × 10^−08^	6.5200 × 10^+01^	**0.0000 × 10^+00^**
*F*13	Mean	8.7058 × 10^+00^	2.5170 × 10^+76^	5.3300 × 10^−03^	5.4671 × 10^−05^	7.6400 × 10^−12^	8.8824 × 10^−16^
	Std	1.2778 × 10^+00^	1.1750 × 10^+77^	7.4800 × 10^−03^	2.6163 × 10^−05^	6.9400 × 10^−12^	**0.0000 × 10^+00^**
*F*14	Mean	9.9800 × 10^−01^	6.3350 × 10^−01^	1.1512 × 10^−03^	9.1182 × 10^−11^	1.9000 × 10^−10^	**0.0000 × 10^+00^**
	Std	5.6000 × 10^−16^	8.6912 × 10^−01^	9.3600 × 10^−04^	7.6752 × 10^−11^	4.3400 × 10^−10^	**0.0000 × 10^+00^**
*F*15	Mean	6.1300 × 10^−03^	4.8452 × 10^−04^	4.5600 × 10^+01^	5.5400 × 10^−16^	2.5100 × 10^+01^	**0.0000 × 10^+00^**
	Std	5.3700 × 10^−03^	6.6000 × 10^−04^	1.1100 × 10^+01^	1.5300 × 10^−16^	6.5200 × 10^+01^	**0.0000 × 10^+00^**
*F*16	Mean	7.7620 × 10^−01^	−1.9400 × 10^−00^	4.7700 × 10^−02^	1.0210 × 10^+01^	1.1700 × 10^+01^	**1.3780 × 10^−05^**
	Std	2.0844 × 10^−01^	5.4200 × 10^−07^	6.5800 × 10^−02^	7.3521 × 10^+00^	2.6600 × 10^+00^	1.4504 × 10^−02^
*F*17	Mean	**4.6481 × 10^+01^**	1.8713 × 10^+03^	1.6900 × 10^+06^	6.7600 × 10^+08^	1.0900 × 10^+09^	3.2042 × 10^+04^
	Std	**6.7914 × 10^+02^**	8.0654 × 10^+05^	1.3200 × 10^+06^	2.6100 × 10^+05^	8.1600 × 10^+10^	2.3582 × 10^+03^
*F*18	Mean	3.0000 × 10^+00^	7.1000 × 10^+05^	5.3300 × 10^+05^	2.7110 × 10^−01^	7.6400 × 10^+12^	**2.0000 × 10^−01^**
	Std	**0.0000 × 10^+00^**	8.6942 × 10^−05^	7.4800 × 10^+05^	1.5000 × 10^−01^	6.9400 × 10^+12^	2.0940 × 10^−07^

**Table 5:** The results are compared with those of three other BOA versions on benchmark functions.

Function		IBOA [33]	HPSO-BOA [34]	CBOA [34]	QLBOA
*F*1	Mean	1.6100 × 10^−30^	3.7400 × 10^−104^	1.0100 × 10^−13^	**0.0000 × 10^+00^**
	Std	3.9000 × 10^−30^	2.0500 × 10^−103^	2.1100 × 10^−13^	**0.0000 × 10^+00^**
*F*2	Mean	5.1100 × 10^−19^	2.6300 × 10^−22^	1.2500 × 10^−14^	**8.0212 × 10^−231^**
	Std	1.7300 × 10^−18^	1.4400 × 10^−21^	2.1500 × 10^−14^	**0.0000 × 10^+00^**
*F*3	Mean	6.1500 × 10^−31^	3.0400 × 10^−71^	6.3000 × 10^−13^	**0.0000 × 10^+00^**
	Std	1.1600 × 10^−30^	1.6700 × 10^−70^	1.3700 × 10^−12^	**0.0000 × 10^+00^**
*F*4	Mean	1.3600 × 10^−19^	3.6100 × 10^−46^	2.7700 × 10^−10^	**1.3380 × 10^−249^**
	Std	1.9700 × 10^−19^	1.9700 × 10^−45^	2.9600 × 10^−10^	**0.0000 × 10^+00^**
*F*5	Mean	2.8900 × 10^+01^	2.9000 × 10^+01^	2.8700 × 10^+01^	**4.7112 × 10^−02^**
	Std	3.4000 × 10^−02^	8.1800 × 10^−02^	**1.3900 × 10^−05^**	5.2924 × 10^−02^
*F*6	Mean	4.4400 × 10^+00^	4.1700 × 10^−02^	**8.5000 × 10^−06^**	3.6750 × 10^−03^
	Std	8.7000 × 10^−01^	6.4000 × 10^−02^	**1.0600 × 10^−05^**	3.0320 × 10^−03^
*F*7	Mean	1.2200 × 10^−04^	2.5500 × 10^−04^	2.0000 × 10^−03^	**1.1774 × 10^−04^**
	Std	8.0600 × 10^−05^	4.0000 × 10^−04^	7.8900 × 10^−04^	**1.1000 × 10^−04^**
*F*8	Mean	8.4500 × 10^−31^	7.1500 × 10^−15^	**2.2400 × 10^−23^**	5.5810 × 10^−02^
	Std	2.5200 × 10^−30^	3.9200 × 10^−14^	**7.5100 × 10^−23^**	2.8200 × 10^−01^
*F*9	Mean	1.3200 × 10^−36^	3.1900 × 10^−118^	6.5800 × 10^−15^	**0.0000 × 10^+00^**
	Std	4.5900 × 10^−36^	1.6800 × 10^−117^	1.1900 × 10^−14^	**0.0000 × 10^+00^**
*F*10	Mean	1.1000 × 10^−30^	3.6400 × 10^−78^	2.3700 × 10^−14^	**0.0000 × 10^+00^**
	Std	2.9000 × 10^−30^	1.9900 × 10^−77^	4.2400 × 10^−14^	**0.0000 × 10^+00^**
*F*11	Mean	**0.0000 × 10^+00^**	1.3200 × 10^−136^	1.5400 × 10^−18^	**0.0000 × 10^+00^**
	Std	**0.0000 × 10^+00^**	6.8400 × 10^−135^	2.8500 × 10^−18^	**0.0000 × 10^+00^**
*F*12	Mean	**0.0000 × 10^+00^**	**0.0000 × 10^+00^**	**0.0000 × 10^+00^**	**0.0000 × 10^+00^**
	Std	**0.0000 × 10^+00^**	**0.0000 × 10^+00^**	**0.0000 × 10^+00^**	**0.0000 × 10^+00^**
*F*13	Mean	8.2400 × 10^−12^	8.6900 × 10^−11^	1.8400 × 10^−09^	**8.8824 × 10^−16^**
	Std	**0.0000 × 10^+00^**	4.7300 × 10^−10^	1.7600 × 10^−09^	**0.0000 × 10^+00^**
*F*14	Mean	**0.0000 × 10^+00^**	**0.0000 × 10^+00^**	1.7000 × 10^−14^	**0.0000 × 10^+00^**
	Std	**0.0000 × 10^+00^**	**0.0000 × 10^+00^**	1.8200 × 10^−14^	**0.0000 × 10^+00^**
*F*15	Mean	**0.0000 × 10^+00^**	**0.0000 × 10^+00^**	2.5700 × 10^−22^	**0.0000 × 10^+00^**
	Std	**0.0000 × 10^+00^**	**0.0000 × 10^+00^**	2.2300 × 10^−24^	**0.0000 × 10^+00^**
*F*16	Mean	9.8300 × 10^+00^	7.2800 × 10^−02^	4.3500 × 10^−04^	**1.3780 × 10^−05^**
	Std	2.4700 × 10^+00^	1.8700 × 10^−01^	**4.6600 × 10^−04^**	1.0504 × 10^−02^
*F*17	Mean	5.8500 × 10^+06^	5.8500 × 10^+04^	2.4500 × 10^+07^	**3.2042 × 10^+04^**
	Std	3.2400 × 10^+05^	7.6200 × 10^+03^	3.2400 × 10^+05^	**2.3582 × 10^+03^**
*F*18	Mean	6.1000 × 10^+04^	4.5300 × 10^+02^	3.6800 × 10^+03^	**2.0000 × 10^−01^**
	Std	5.2000 × 10^+03^	3.1200 × 10^+01^	4.2700 × 10^+03^	**2.3640 × 10^−07^**

Note: The best results are shown in bold.

**Table 6:** The Wilcoxon results of comparison algorithms.

No.	PSO	GA	DE	ABC	BOA	CBOA	IBOA	HPSOBOA
*F*1	1.2118 × 10^−12^	1.2118 × 10^−12^	1.2118 × 10^−12^	1.2118 × 10^−12^	1.2118 × 10^−12^	3.0345 × 10^−11^	1.2118 × 10^−12^	1.2118 × 10^−12^
*F*2	3.0199 × 10^−11^	2.9802 × 10^−11^	3.0199 × 10^−11^	3.0199 × 10^−11^	3.0199 × 10^−11^	3.0199 × 10^−11^	3.0199 × 10^−11^	3.0199 × 10^−11^
*F*3	1.2118 × 10^−12^	1.2118 × 10^−12^	1.2118 × 10^−12^	1.2118 × 10^−12^	1.2118 × 10^−12^	2.1449 × 10^−13^	1.2118 × 10^−12^	1.2118 × 10^−12^
*F*4	3.0199 × 10^−11^	3.0212 × 10^−11^	3.0199 × 10^−11^	3.0199 × 10^−11^	3.0199 × 10^−11^	3.0199 × 10^−11^	3.0199 × 10^−11^	3.0199 × 10^−11^
*F*5	3.4742 × 10^−10^	3.9935 × 10^−04^	3.9881 × 10^−04^	3.0199 × 10^−11^	3.0199 × 10^−11^	**3.1559 × 10^−01^**	8.1527 × 10^−11^	8.5641 × 10^−04^
*F*6	5.9673 × 10^−09^	2.8745 × 10^−10^	3.0199 × 10^−11^	1.3685 × 10^−05^	3.0199 × 10^−11^	3.3384 × 10^−11^	1.3289 × 10^−10^	3.0199 × 10^−11^
*F*7	3.0199 × 10^−11^	3.0199 × 10^−11^	3.0199 × 10^−11^	3.0199 × 10^−11^	3.0199 × 10_−11_	1.0139 × 10^−10^	1.9963 × 10^−05^	3.0199 × 10^−11^
*F*8	1.2118 × 10^−12^	1.2118 × 10^−12^	1.2118 × 10^−12^	1.2118 × 10^−12^	1.2118 × 10^−12^	7.6083 × 10^−13^	1.2118 × 10^−12^	1.2118 × 10^−12^
*F*9	1.2118 × 10^−12^	1.2118 × 10^−12^	1.2118 × 10^−12^	1.2118 × 10^−12^	1.2118 × 10^−12^	**2.3371 × 10^−01^**	1.2118 × 10^−12^	1.2118 × 10^−12^
*F*10	1.2118 × 10^−12^	3.0199 × 10^−11^	1.2118 × 10^−12^	1.2118 × 10^−12^	1.2118 × 10^−12^	3.3735 × 10^−02^	1.2118 × 10^−12^	1.2118 × 10^−12^
*F*11	1.2118 × 10^−12^	1.2118 × 10^−12^	1.2118 × 10^−12^	1.2118 × 10^−12^	1.2118 × 10^−12^	5.6493 × 10^−13^	1.2118 × 10^−12^	1.2118 × 10^−12^
*F*12	1.9324 × 10^−09^	1.2118 × 10^−12^	1.2118 × 10^−12^	1.2118 × 10^−12^	1.2118 × 10^−12^	4.5336 × 10^−12^	3.9229 × 10^−05^	2.2574 × 10^−04^
*F*13	1.2118 × 10^−12^	1.2118 × 10^−12^	1.2118 × 10^−12^	1.2118 × 10^−12^	1.2118 × 10^−12^	1.2118 × 10^−12^	3.0199 × 10^−11^	1.2118 × 10^−12^
*F*14	1.2118 × 10^−12^	3.0199 × 10^−11^	1.2118 × 10^−12^	1.2118 × 10^−12^	1.2118 × 10^−12^	4.3492 × 10^−12^	2.5474 × 10^−04^	1.2118 × 10^−12^
*F*15	1.2118 × 10^−12^	1.2118 × 10^−12^	1.2118 × 10^−12^	1.2118 × 10^−12^	1.2118 × 10^−12^	1.2118 × 10^−12^	1.2118 × 10^−12^	1.2118 × 10^−12^
*F*16	6.7869 × 10^−02^	3.0199 × 10^−11^	1.8577 × 10^−01^	3.0199 × 1^0−11^	3.0199 × 10^−11^	3.1589 × 10^−10^	1.3252 × 10^−06^	3.0199 × 10^−11^
*F*17	1.5369 × 10^−03^	3.0199 × 10^−11^	**1.366 × 10^−02^**	3.5350 × 10^−09^	5.6900 × 10^−08^	1.1549 × 10^−12^	1.4333 × 10^−05^	2.0149 × 10^−03^
*F*18	1.6490 × 10^−03^	3.0199 × 10^−11^	2.5455 × 10^−05^	**4.3230 × 10^−01^**	1.2118 × 10^−12^	1.2118 × 10^−12^	3.8406 × 10^−03^	1.2118 × 10^−12^

Note: Bold type indicates values > 0.05.

**Table 7:** Comparison of the results of the QLBOA with others on the CEC2022 test suite.

Function		PSO [39]	ACO [40]	ABC [41]	DE [39]	SCA [42]	SOA [42]	BOA [43]	QLBOA
*F*1	Mean	1.9400 × 10^+03^	1.7000 × 10^+03^	1.3900 × 10^+03^	2.4164 × 10^+03^	1.2745 × 10^+03^	1.1900 × 10^+03^	7.9116 × 10^+03^	**3.1289 × 10^+02^**
	Std	7.0900 × 10^+02^	5.7400 × 10^+02^	2.7200 × 10^+02^	2.7820 × 10^+02^	6.4150 × 10^+02^	1.8300 × 10^+02^	3.2110 × 10^+03^	**5.8670 × 10^+01^**
*F*2	Mean	9.8800 × 10^+02^	5.4900 × 10^+02^	4.2200 × 10^+02^	2.5130 × 10^+02^	4.6409 × 10^+02^	4.0000 × 10^+03^	4.3443 × 10^+03^	**4.1452 × 10^+02^**
	Std	1.6800 × 10^+02^	3.9700 × 10^+02^	1.4700 × 10^+02^	**7.0540 × 10^+00^**	2.4199 × 10^+01^	1.4800 × 10^+03^	4.6354 × 10^+02^	2.5278 × 10^+01^
*F*3	Mean	1.5300 × 10^ +03^	1.3400 × 10^+03^	7.2100 × 10^+02^	7.3080 × 10^+02^	6.1885 × 10^+03^	9.3100 × 10^+02^	1.3365 × 10^+03^	**7.1135 × 10^+02^**
	Std	4.5200 × 10^ +01^	5.0100 × 10^+01^	**1.0300 × 10^+01^**	7.6000 × 10^+01^	4.9100 × 10^+01^	4.9900 × 10^+01^	7.9134 × 10^+01^	4.6948 × 10^+01^
*F*4	Mean	1.8200 × 10^+03^	1.6500 × 10^+03^	1.0300 × 10^+03^	2.6500 × 10^+03^	1.6540 × 10^+03^	1.2400 × 10^+03^	1.6562 × 10^+03^	**1.0012 × 10^+03^**
	Std	6.4400 × 10^+01^	5.5400 × 10^+01^	1.2300 × 10^+01^	**4.3370 × 10^+00^**	4.3850 × 10^+01^	4.9700 × 10^+01^	4.6885 × 10^+01^	4.8552 × 10^+01^
*F*5	Mean	7.7600 × 10^ +03^	4.7900 × 10^+03^	1.5000 × 10^+02^	**7.4620 × 10^−02^**	4.4750 × 10^+04^	2.0700 × 10^+03^	4.3724 × 10^+03^	9.1416 × 10^+02^
	Std	1.2200 × 10^ +02^	3.6200 × 10^ +03^	3.0100 × 10^+02^	8.9320 × 10^−02^	8.6800 × 10^+01^	4.5200 × 10^+02^	4.9864 × 10^+03^	**7.4438 × 10^+01^**
*F*6	Mean	2.5700 × 10^+03^	2.4900 × 10^ +03^	2.2500 × 10^+03^	2.9460 × 10^+03^	2.5000 × 10^+04^	6.5800 × 10^+03^	2.5074 × 10^+04^	**2.0000 × 10^+03^**
	Std	4.6200 × 10^+02^	2.8900 × 10^+03^	5.1100 × 10^+02^	5.7010 × 10^+02^	2.1400 × 10^+03^	2.6000 × 10^+03^	4.8867 × 10^+03^	**2.7954 × 10^+02^**
*F*7	Mean	4.6900 × 10^+03^	4.1700 × 10^+03^	3.0800 × 10^+03^	1.1360 × 10^+03^	2.7405 × 10^+03^	1.0100 × 10^+04^	4.7423 × 10^+03^	**2.0962 × 10^+03^**
	Std	1.8700 × 10^+02^	1.4600 × 10^+02^	1.0500 × 10^+02^	5.5810 × 10^+02^	6.2500 × 10^+02^	4.1500 × 10^+03^	4.2523 × 10^+02^	**8.0452 × 10^+01^**
*F*8	Mean	3.6400 × 10^ +03^	3.1800 × 10^+03^	2.3300 × 10^+03^	7.7570 × 10^+03^	2.6505 × 10^+03^	**2.2000 × 10^+02^**	3.3674 × 10^+03^	2.6948 × 10^+03^
	Std	1.2600 × 10^+02^	3.2300 × 10^+01^	1.0600 × 10^+01^	5.1360 × 10^+01^	9.3450 × 10^+01^	3.9300 × 10^+03^	9.3323 × 10^+01^	**1.0420 × 10^+01^**
*F*9	Mean	5.4300 × 10^ +03^	4.2400 × 10 ^+03^	2.8400 × 10^+03^	**2.1240 × 10^+03^**	5.1550 × 10^+03^	3.4100 × 10^+02^	5.1562 × 10^+03^	2.6297 × 10^+03^
	Std	3.7800 × 10^ +02^	9.4900 × 10^+01^	1.4000 × 10^+01^	**2.9450 × 10^−02^**	1.4700 × 10^+01^	3.5800 × 10^+03^	2.4776 × 10^+02^	9.9538 × 10^+00^
*F*10	Mean	6.8700 × 10^+03^	4.6700 × 10 ^+03^	3.0600 × 10^+03^	3.2000 × 10^+03^	3.7804 × 10^+03^	6.3400 × 10^+03^	5.7883 × 10^+03^	**3.0014 × 10^+03^**
	Std	2.9500 × 10^+02^	9.6300 × 10 ^+01^	**1.0300 × 10^+01^**	1.2870 × 10^+01^	4.3100 × 10^+02^	6.8000 × 10^+03^	4.3124 × 10^+02^	1.4200 × 10^+02^
*F*11	Mean	3.0600 × 10^+04^	1.7100 × 10^+04^	6.6400 × 10^+05^	3.9200 × 10^+02^	1.6450 × 10^+04^	**3.2900 × 10^+02^**	1.6478 × 10^+04^	2.9350 × 10^+03^
	Std	2.9300 × 10^+03^	1.7400 × 10^+03^	1.8300 × 10^+05^	7.9870 × 10^+01^	3.4607 × 10^+01^	4.3600 × 10^+03^	1.0234 × 10^+03^	**3.0226 × 10^+01^**
*F*12	Mean	2.8100 × 10^+04^	1.5500 × 10^+04^	5.5700 × 10^+03^	**1.0350 × 10^+00^**	1.8400 × 10^+04^	1.6300 × 10^+03^	1.8402 × 10^+04^	7.6228 × 10^+03^
	Std	1.9200 × 10^+03^	8.3500 × 10^+02^	9.4400 × 10^+01^	**1.8570 × 10^−02^**	5.5370 × 10^+02^	1.1300 × 10^+04^	6.5305 × 10^+02^	1.7782 × 10^+03^

**Table 8:** The Wilcoxon results of comparison algorithms on the CEC2022 suite.

No.	PSO	ACO	ABC	DE	SCA	SOA	BOA
*F*1	3.0199 × 10^−11^	3.0199 × 10^−11^	3.0199 × 10^−11^	3.5105 × 10^−08^	3.0199 × 10^−11^	3.5384 × 10^−11^	3.0199 × 10^−11^
*F*2	8.6634 × 10^−05^	3.0199 × 10^−11^	2.3168 × 10^−06^	1.1058 × 10^−04^	3.0199 × 10^−11^	2.7829 × 10^−07^	3.0199 × 10^−11^
*F*3	3.8347 × 10^−05^	3.0199 × 10^−11^	6.4878 × 10^−09^	**5.3874 × 10^−02^**	3.0199 × 10^−11^	3.0199 × 10^−11^	3.8507 × 10^−05^
*F*4	3.0199 × 10^−11^	3.0199 × 10^−11^	3.0199 × 10^−11^	4.4645 × 10^−08^	3.4384 × 10^−11^	5.4541 × 10^−11^	3.0199 × 10^−11^
*F*5	2.3715 × 10^−10^	3.0199 × 10^−11^	3.5201 × 10^−07^	3.0199 × 10^−11^	3.0199 × 10^−11^	4.6159 × 10^−10^	4.0772 × 10^−11^
*F*6	3.4642 × 10^−10^	4.3374 × 10^−02^	2.6947 × 10^−09^	2.5771 × 10^−07^	8.6334 × 10^−05^	3.4542 × 10^−10^	3.0199 × 10^−11^
*F*7	3.5923 × 10^−05^	5.4941 × 10^−11^	**3.4029 × 10^−01^**	4.0772 × 10^−11^	6.0658 × 10^−11^	1.6813 × 10^−04^	2.3168 × 10^−06^
*F*8	3.0199 × 10^−11^	8.1714 × 10^−10^	3.0199 × 10^−11^	3.0199 × 10^−11^	5.7941 × 10^−11^	3.6597 × 10^−11^	3.0199 × 10^−11^
*F*9	4.4440 × 10^−07^	3.0199 × 10^−11^	8.1975 × 10^−07^	**2.3399 × 10^−01^**	3.0199 × 10^−11^	1.9883 × 10^−02^	3.0199 × 10^−11^
*F*10	1.4743 × 10^−10^	1.6480 × 10^−08^	7.3391 × 10^−11^	3.3374 × 10^−11^	8.4348 × 10^−09^	1.4810 × 10^−09^	1.4643 × 10^−10^
*F*11	1.9527 × 10^−03^	3.0199 × 10^−11^	**4.5530 × 10^−01^**	2.7829 × 10^−07^	3.0199 × 10^−11^	3.4783 × 10^−01^	3.0199 × 10^−11^
*F*12	3.0199 × 10^−11^	3.0199 × 10^−11^	3.0199 × 10^−11^	3.0199 × 10^−11^	3.0199 × 10^−11^	3.0199 × 10^−11^	3.0199 × 10^−11^

Note: Bold type indicates values > 0.05.

**Table 9:** Model symbols and parameter settings.

Symbol	Meaning
*N*	Set of nodes, *N* = {0, 1, …, n}
*N’*	Customer collection
*K*	Set k of distribution vehicles, *k* ∈ K
*Q*	Maximum vehicle loading capacity
*t_i_*	Customer delivery time *i* ∈ *N*
*q_ijk_*	Load of vehicle *k* from customer *i* to customer *j*
[*ET_i_*, *DT_i_*, *LT_i_*]	The service time window at customer point *i*
*δ_e_*	Waiting penalty for early arrival of customer *i*
*δ_li_*	Tardiness penalty for late arrival of customer *i*
*d_ij_*	The distance from customer point *i* to *j*
*f_ijk_*	Fuel consumption rate of vehicle *k* on road segment (*i*, *j*) (kg/km)
*C_v_*	Unit fuel consumption cost (CNY/L)
*e_ijk_*	Carbon emission rate of vehicle *k* on road segment (*i*, *j*) (kg/km)
*C_k_*	Unit transportation cost (CNY/km)
*t_ijk_*	Travel time of vehicle *k* on road segment (*i*, *j*)
*C_f_*	Charge per unit of carbon emissions (CNY/kg)
*C_e_*	Vehicle fixed cost (CNY/car)
*v_k_*	The traveling speed of vehicle *k*
*ε*	Customer personal preference value
*M*	Total vehicle weight (kg)
*g*	Constant of gravity (9.81 m/s^2^)
*ζ*	Speed of engine
*V*	Displacement of engine
*ξ*	Diesel fuel calorific value
*x_ijk_*	0–1 variable, which is 1 if vehicle *k* is driving on road (*i*, *j*) and 0 otherwise
*y_ik_*	0–1 variable, 1 when customer point *i* is served by vehicle *k* and 0 otherwise
*z_k_*	0–1 variable, 1 when vehicle *k* is used and 0 otherwise

**Table 10:** Problem parameter settings.

Symbol	Meaning
*K*	25
Coefficient of penalty *δ*	100
Unit transportation cost *C_k_* (CNY/km)	1
Unit fuel consumption cost *C_v_* (CNY/L)	7.5
Charge per unit of carbon emissions *C_f_* (CNY/kg)	0.0528
Vehicle fixed cost *C_e_* (CNY/car)	100
*ω*_0_, *ω*_1_, *ω*_2_, *ω*_3_, *ω*_4_, *ω*_5_, *ω*_6_	110, 0, −0.0011, −0.00235, 0, 0
*χ*_0_, *χ*_1_, *χ*_2_, *χ*_3_, *χ*_4_, *χ*_5_, *χ*_6_, *χ*_7_	1.27, 0.0614, 0, −0.0011, −0.00235, 0, 0, −1.33

**Table 11:** Analysis of the influence of the subjective preferences of decision makers on three objectives.

Datasets	*ε*	Transportation Cost	Fuel Cost	Penalty Cost	Number of Vehicles
C107	0.2	972.85	1687.28	256.42	8
	0.6	987.59	1258.63	278.36	11
	0.8	1008.35	1381.44	112.05	13
C202	0.2	916.14	1118.112	236.12	7
	0.6	909.76	1048.52	208.14	10
	0.8	1193.26	1634.77	125.02	11
R106	0.2	1185.45	1607.34	308.25	11
	0.6	1199.10	1642.16	225.03	14
	0.8	1346.10	1844.26	175.74	15
R201	0.2	1206.68	1652.32	227.64	6
	0.6	988.85	1353.29	198.76	9
	0.8	1326.53	1817.48	155.31	11
RC102	0.2	1695.36	2322.64	198.52	14
	0.6	1685.74	2135.84	108.43	16
	0.8	1702.84	2331.78	89.35	17
RC206	0.2	1589.37	2176.93	225.35	6
	0.6	1466.67	1906.07	208.93	10
	0.8	1697.85	2324.8	205.35	12

**Table 12:** Analysis of the influence of weight factors on three objectives.

Datasets	Weighting Factor (*λ*)	Transportation Cost	Fuel Cost	Penalty Cost	Number of Vehicles
C107	*λ*_1_ = 0.6, *λ*_2_ = 0.3, *λ*_3_ = 0.1	395.04	881.04	250.52	8
	*λ*_1_ = 0.1, *λ*_2_ = 0.6, *λ*_3_ = 0.3	888.83	503.45	194.85	11
	*λ*_1_ = 0.3, *λ*_2_ = 0.1, *λ*_3_ = 0.6	691.31	1132.77	111.34	13
C202	*λ*_1_ = 0.6, *λ*_2_ = 0.3, *λ*_3_ = 0.1	363.90	733.96	187.33	8
	*λ*_1_ = 0.1, *λ*_2_ = 0.6, *λ*_3_ = 0.3	818.78	419.41	145.70	10
	*λ*_1_ = 0.3, *λ*_2_ = 0.1, *λ*_3_ = 0.6	636.83	943.67	83.26	12
R106	*λ*_1_ = 0.6, *λ*_2_ = 0.3, *λ*_3_ = 0.1	479.64	1149.51	202.53	12
	*λ*_1_ = 0.1, *λ*_2_ = 0.6, *λ*_3_ = 0.3	1079.19	656.86	157.52	14
	*λ*_1_ = 0.3, *λ*_2_ = 0.1, *λ*_3_ = 0.6	839.37	1477.94	90.01	15
R201	*λ*_1_ = 0.6, *λ*_2_ = 0.3, *λ*_3_ = 0.1	395.54	947.30	178.88	6
	*λ*_1_ = 0.1, *λ*_2_ = 0.6, *λ*_3_ = 0.3	889.97	541.32	139.13	9
	*λ*_1_ = 0.3, *λ*_2_ = 0.1, *λ*_3_ = 0.6	692.20	1217.96	79.50	12
RC102	*λ*_1_ = 0.6, *λ*_2_ = 0.3, *λ*_3_ = 0.1	674.30	1495.09	97.59	11
	*λ*_1_ = 0.1, *λ*_2_ = 0.6, *λ*_3_ = 0.3	1517.17	854.34	75.90	13
	*λ*_1_ = 0.3, *λ*_2_ = 0.1, *λ*_3_ = 0.6	1180.02	1922.26	43.37	17
RC206	*λ*_1_ = 0.6, *λ*_2_ = 0.3, *λ*_3_ = 0.1	586.66	1334.25	188.04	6
	*λ*_1_ = 0.1, *λ*_2_ = 0.6, *λ*_3_ = 0.3	1319.99	762.43	146.25	10
	*λ*_1_ = 0.3, *λ*_2_ = 0.1, *λ*_3_ = 0.6	1026.66	1715.46	83.57	12

**Table 13:** Comparison of three objectives for QLBOA and three algorithms.

Datasets	Algorithms	Transportation Cost	Fuel Cost	Penalty Cost
C107	GA	449.04	1081.05	270.53
	ACO	435.87	1103.47	247.95
	BOA	691.31	1132.77	311.34
	QLBOA	395.04	881.04	250.52
C202	GA	347.68	678.78	217.63
	ACO	818.78	579.46	145.70
	BOA	635.83	973.68	285.76
	QLBOA	363.90	733.96	187.33
R106	GA	426.57	1648.75	237.47
	ACO	1079.19	656.86	257.52
	BOA	839.37	1477.94	390.01
	QLBOA	479.64	1149.51	202.53
R201	GA	405.55	997.39	190.08
	ACO	889.97	841.32	139.13
	BOA	692.20	1217.96	79.50
	QLBOA	395.54	947.30	178.88
RC102	GA	1078.38	1585.89	108.96
	ACO	1017.17	1064.04	105.90
	BOA	1180.02	1906.27	243.37
	QLBOA	674.30	1495.09	97.59
RC206	GA	1088.67	1054.25	204.33
	ACO	1386.34	1056.49	246.25
	BOA	1056.47	1895.44	283.57
	QLBOA	586.66	1334.25	188.04

## Data Availability

The original contributions presented in the study are included in the article. Further inquiries can be directed to the corresponding authors.
